# IMGT^®^ Nomenclature of Engineered IGHG Variants Involved in Antibody Effector Properties and Formats

**DOI:** 10.3390/antib11040065

**Published:** 2022-10-18

**Authors:** Marie-Paule Lefranc, Gérard Lefranc

**Affiliations:** IMGT®, The International ImMunoGeneTics Information System®, Laboratoire d’ImmunoGénétique Moléculaire (LIGM), Institut de Génétique Humaine (IGH), Centre National de la Recherche Scientifique (CNRS), Université de Montpellier (UM), UMR 9002 CNRS-UM, CEDEX 5, 34396 Montpellier, France

**Keywords:** IMGT, immunogenetics, immunoinformatics, immunoglobulin (IG), antibody, system biology, bioengineering, allotypes, variants, effector properties

## Abstract

The constant region of the immunoglobulin (IG) or antibody heavy gamma chain is frequently engineered to modify the effector properties of the therapeutic monoclonal antibodies. These variants are classified in regards to their effects on effector functions, antibody-dependent cytotoxicity (ADCC), antibody-dependent phagocytosis (ADCP), complement-dependent cytotoxicity (CDC) enhancement or reduction, B cell inhibition by the coengagement of antigen and FcγR on the same cell, on half-life increase, and/or on structure such as prevention of IgG4 half-IG exchange, hexamerisation, knobs-into-holes and the heteropairing H-H of bispecific antibodies, absence of disulfide bridge inter H-L, absence of glycosylation site, and site-specific drug attachment engineered cysteine. The IMGT engineered variant identifier is comprised of the species and gene name (and eventually allele), the letter ‘v’ followed by a number (assigned chronologically), and for each concerned domain (e.g, CH1, h, CH2 and CH3), the novel AA (single letter abbreviation) and IMGT position according to the IMGT unique numbering for the C-domain and between parentheses, the Eu numbering. IMGT engineered variants are described with detailed amino acid changes, visualized in motifs based on the IMGT numbering bridging genes, sequences, and structures for higher order description.

## 1. Introduction

The adaptive immune response, acquired by jawed vertebrates (or *gnathostomata*) more than 450 million years ago and found in all extant jawed vertebrate species from fish to humans, is characterized by a remarkable immune specificity and memory, which are the properties of the B and T cells because of the extreme diversity of their antigen receptors [1]. The antigen receptors of the adaptive immune response [1,2] comprise the immunoglobulins (IG) or antibodies of the B cells and plasmocytes [3,4] and the T cell receptors (TR) of the T cells [5]. The IG recognizes antigens in their native (unprocessed) form, whereas the TR recognizes processed antigens, which are presented as peptides through its highly polymorphic major histocompatibility (MH, in humans HLA for human leucocyte antigens) proteins [6]. Immunoglobulins (IG) or antibodies serve a dual role in immunity. First, they both recognize antigens on the surface of foreign bodies such as bacteria and viruses, and second, they trigger elimination mechanisms such as cell lysis and phagocytosis to rid the body of these invading cells and particles [4]. IMGT^®^, the international ImMunoGeneTics information system^®^ (https://www.imgt.org) (accessed on 11 October 2022) [1], was created in 1989 by Marie-Paule Lefranc in Montpellier, France, Laboratoire d’ImmunoGénétique Moléculaire (LIGM) des Prof G. and M-P. Lefranc (Université de Montpellier and CNRS) to manage the huge diversity of the IG and TR repertoires. For the first time, immunoglobulin (IG) or antibody and T cell receptor (TR) variable (V), diversity (D), joining (J) and constant (C) genes were officially recognized as ‘genes’ and conventional genes [1,3,5,7,8,9,10]. Through its creation, IMGT^®^ marks the advent of a new science, immunoinformatics, which emerged at the interface between immunogenetics and bioinformatics [1]. As an ontology and system, IMGT^®^ bridges genes, sequences and structures of the antigen receptors to better understand their functions. Focusing on the constant region of the IgG, a standardized definition of engineered variants of therapeutic antibodies is provided based on the IMGT concepts.

## 2. An Ontology and a System to Bridge Genes, Sequences and Structures to Functions

**Figure 1:**
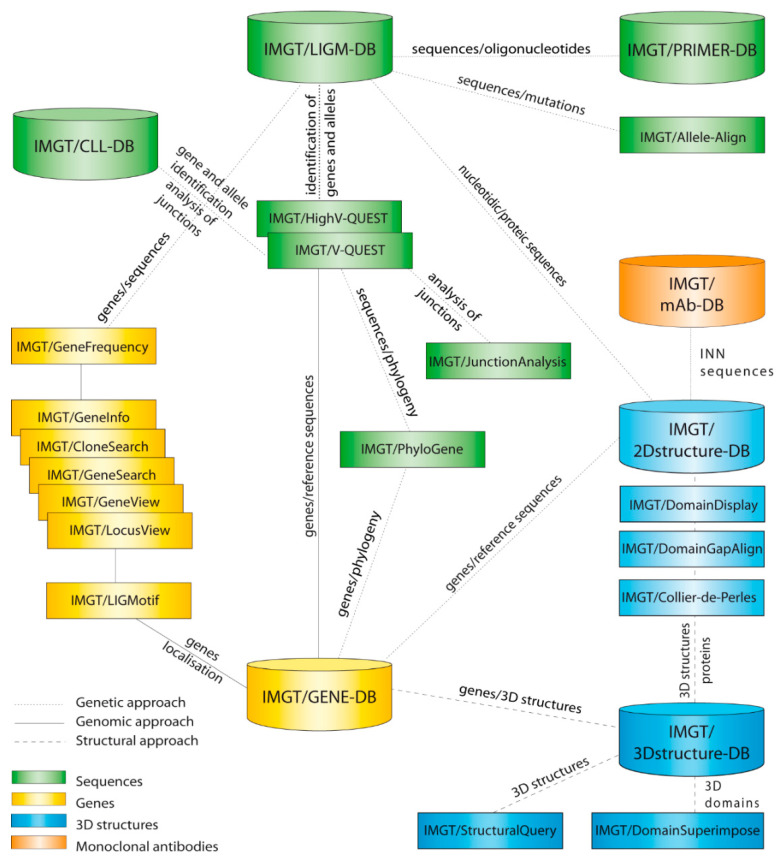
IMGT^®^ is the international ImMunoGenetics information system^®^ (https://www.imgt.org) [11,12,13,14,15,16,17,18,19,20,21]. The IMGT web resources (>25,000 pages, the IMGT Marie-Paule page) are not shown. IMGT/mAb-DB, the interface for therapeutic monoclonal antibodies and fusion proteins for immune applications (FPIA), has been available online since 4 December 2009 and IMGT/HighV-QUEST portal for the next generation sequencing (NGS) high-throughput sequence analysis since 22 November 2010 (with permission from M-P.Lefranc and G. Lefranc, LIGM, Founders of IMGT^®^ from the international ImMunoGeneTics information system^®^ (https://www.imgt.org)).

IMGT^®^, the international ImMunoGeneTics information system^®^ (Figure 1) [1,11,12,13,14,15,16,17,18,19,20,21], is an integrated system for the genes, sequences and structures of the IG or antibodies, TR and MH of the adaptive immune responses of the jawed vertebrates, as well as other proteins of the IG superfamily (IgSF) [22] and MH superfamily (MhSF) of vertebrates and invertebrates [23].

Immunoinformatics [1] builds and organizes molecular immunogenetics knowledge to be managed and shared in IMGT^®^. IMGT**^®^** comprises seven databases [24,25,26,27,28,29,30], 17 tools [31,32,33,34,35,36,37,38,39,40,41,42,43,44,45,46,47,48,49,50] and more than 25,000 pages of web resources (Table 1). IMGT**^®^** dababases are specialized in sequences (i.e., IMGT/LIGM-DB [24,25]), genes and alleles (IMGT/GENE-DB [26]), two-dimensional (2D) structures (IMGT/2Dstructure-DB) and three-dimensional (3D) structures (IMGT/3Dstructure-DB) [27,28,29], whereas the IMGT/mAb-DB [30] interface allows the querying of therapeutic monoclonal antibodies (IG, mAb), fusion proteins for immunological applications (FPIA), composite proteins for clinical applications (CPCA) and related proteins (RPI) of therapeutic interest (with links to amino acid sequences in IMGT/2Dstructure-DB, and if available, to 3D structures in IMGT/3D structure-DB. The IMGT**^®^** tools include: (1) For nucleotide sequence analysis, IMGT/V-QUEST [31,32,33,34,35,36] and the integrated IMGT/JunctionAnalysis [37,38] and IMGT/Automat [39,40] tools, and for next generation sequencing, the high-throughput version IMGT/HighV-QUEST [36,41,42,43,44,45] and the downloadable IMGT/StatClonotype [46,47] package (which allows for statistical pairwise analysis of the diversity and expression of the IMGT clonotypes (AA) [43] and repertoire comparisons in adaptive immune responses); (2) for genomic analysis, IMGT/LIGMotif [48] (which allows for the identification and description of new genes in genomic sequences); (3) for amino acid sequence analysis per the domain, IMGT/DomainGapAlign [28,49,50]; and (4) for graphical representations of the domains, the IMGT/Collier-de-Perles tool [51] (e.g., IMGT Colliers de Perles of the variable (V), constant (C) and groove (G) domains). IMGT^®^ Web resources (‘the IMGT Marie-Paule page’) comprise the IMGT Repertoire (IG and TR, MH and RPI), IMGT Scientific chart, IMGT Education (IMGT Lexique, Aide-mémoire (amino acid physicochemical properties [52], splicing sites) and tutorials, etc.).

The bridging of genes, structures and functions is based on the IMGT-ONTOLOGY axioms and concepts from which were generared the IMGT Scientific chart rules [78,79,80,81,82] (Table 2): CLASSIFICATION for theIMGT standardized gene and allele nomenclature [1,2,3,4,5,7,8,9,10,61,62,63], IDENTIFICATION for IMGT standardized keywords and keyword abbreviations (e.g., clonotype, paratope and epitope, variant, Fc receptor and FcR) [53,54], DESCRIPTION forIMGT standardized labels [55,56,57,58] (e.g., complementarity determining region (CDR)-IMGT (CDR1-IMGT to CDR3-IMGT) [57] and framework region (FR-IMGT) (FR1-IMGT to FR4-IMGT) [58]), NUMEROTATION for the IMGT unique numbering [64,65,66,67,68,69,70,71,72] and the IMGT Colliers de Perles [51,73,74,75,76,77]. IMGT positions per domain are used in Protein displays, Alignments of alleles, CDR-IMGT lengths, Allotypes [59,60] sections of the IMGT Repertoire, and to number amino acids involved in paratope/epitope (antigen receptor V-domains/target interactions [83]) (Table 1) and in effector properties (antigen receptor C-domain/effector binding proteins [6]).

IMGT standards have been used since 2006 in the description of the therapeutic antibodies published in the World Health Organization’s (WHO) International Nonproprietary Names (INN) programme [84,85,86]. Since 2003, IMGT^®^ has been widely used in the analysis of therapeutical antibodies for humanization and/or engineering [4,11,13,87,88,89,90,91,92,93,94,95,96].

## 3. Immunoglobulin IgG Receptor, Chains, Domains and Amino Acids

**Figure 2:**
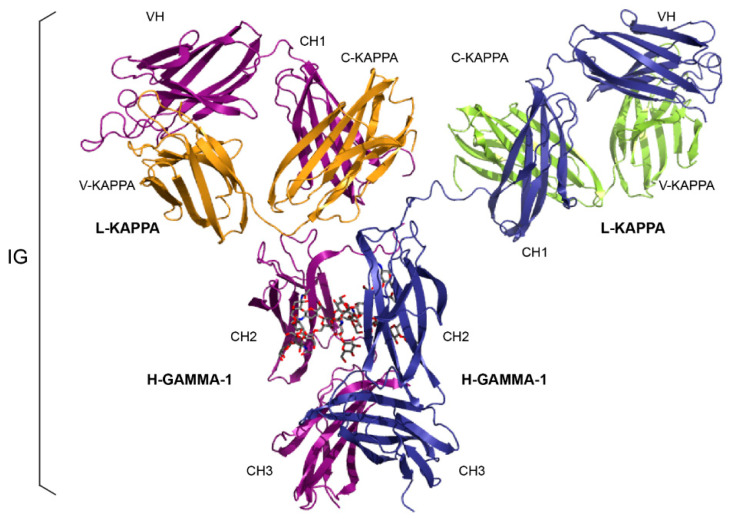
Immunoglobulin IgG1. The structure is that of the antibody b12, an IgG1-kappa, and so far is the only complete human IG crystallized (PDB code: 1hzh, from IMGT^®^ https://www.imgt.org, IMGT/3Dstructure-DB). H-GAMMA-1 and L-KAPPA (usedfor the chains), VH, CH1, CH2, CH3, V-KAPPA and C-KAPPA (for the domains) are written in capital letters as they are IMGT standardized labels (DESCRIPTION) [1]. This first 3D-structure of a complete *Homo sapiens* IG shows the expected Y shape with the two Fragment antigen binding (Fab) arms (one L-KAPPA light chain (V-KAPPA-C-KAPPA) paired to the VH-CH1 of each H-GAMMA-1 heavy chain) and the Fragment crystallisable (Fc), made of the paired hinge-CH2-CH3 of the two H-GAMMA-1 heavy chains. The figure also shows the relative position, in space, of the L-KAPPA relative to the VH-CH1 in each Fab (in the front on the left hand side, and the back right hand side). The sequences of the two H-GAMMA1 chains (colored in purple and dark blue for a better visibility) are identical and the sequences of the two L-KAPPA chains (colored in orange and green for a better visibility) are identical (with permission from M-P. Lefranc and G. Lefranc, LIGM, Founders of IMGT^®^, the international ImMunoGeneTics information system^®^, https://www.imgt.org).

The *Homo sapien’s* IgG1-kappa (Figure 2) is taken as an example (Table 3) because it is the most represented subclass in therapeutic antibodies.

**Figure 3:**
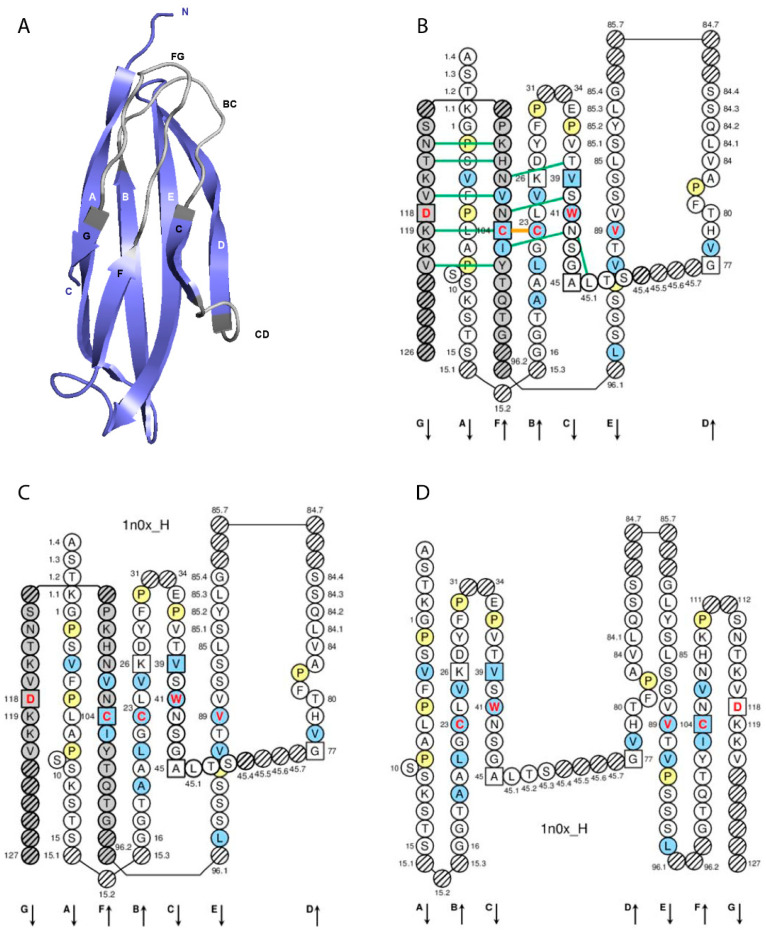
IG constant (**C**) domain. (**A**) 3D structure ribbon representation with the IMGT strand and loop delimitations. (**B**) IMGT Collier de Perles on two layers with hydrogen bonds. The IMGT Colliers de Perles on two layers show, in the forefront, the GFC strands, and in the back, the ABED strands (located at the interface CH1/CL of the IG), linked by the CD transversal strand. The IMGT Collier de Perles with hydrogen bonds (green lines online, only shown here for the GFC sheet) is generated by the IMGT/Collier de Perles tool [51] integrated in the IMGT/3Dstructure-DB, from experimental 3D structure data. (**C**) IMGT Collier de Perles on two layers from IMGT/DomainGapAlign [28,49,50]. (**D**) IMGT Colliers de Perles on one layer. Amino acids are shown in the one-letter abbreviation. All proline (P) are shown online in yellow. IMGT anchors are represented by squares. Hatched circles are IMGT gaps according to the IMGT unique numbering for the C-domain [68]. Positions with bold (online red) letters indicate the four conserved positions that are common to a V-domain and to a C-domain: 23 (1st-CYS), 41 (CONSERVED-TRP), 89 (hydrophobic), 104 (2nd-CYS), and position 118, which is only conserved in V-DOMAIN. The identifier of the chain to which the CH-domain belongs is 1n0x_H (from the *Homo sapiens* b12 Fab, in IMGT/3Dstructure-DB, https://www.imgt.org) [27,28,29]. The 3D ribbon representation was obtained using PyMOL and “IMGT numbering comparison” of 1n0x_H (CH1) from IMGT/3Dstructure-DB (https://www.imgt.org) [27,28,29].

In the IMGT system, the C-domain includes the C-DOMAIN of the IG and of the TR [1] and the C-LIKE-DOMAIN of the IgSF other than IG and TR [22]. The C-domain description of any receptor, any chain and any species is based on the IMGT unique numbering for the C-domain (C-DOMAIN and C-LIKE-DOMAIN) [68]. A C-domain (Figure 3) comprises about 90–100 amino acids and is made up of seven antiparallel beta strands (A, B, C, D, E, F and G), linked by beta turns (AB, DE and EF), a transversal strand (CD) and two loops (BC and FG), and forms a sandwich of two sheets [ABED] [GFC]. A C-domain has a topology and a three-dimensional structure that is similar to that of a V-domain [67], but without the C’ and C’’ strands and the C’C’’ loop, which is replaced by a transversal CD strand [68]. The lengths of the strands and loops (Table 4) are visualized in the IMGT Colliers de Perles on one layer and two layers (Figure 3).

There are six IMGT anchors in a C-domain (four of them identical to those of a V-domain): Positions 26 and 39 (anchors of the BC loop), 45 and 77 (by extension, anchors of the CD strand as there is no C’-C’’ loop in a C-domain [68]), and 104 and 118 (anchors of the FG loop). A C-domain has five characteristic amino acids at given positions (positions with bold (online red) letters in the IMGT Colliers de Perles). Four of them are highly conserved and hydrophobic [52] and are common to the V-domain: 23 (1st-CYS), 41 (CONSERVED-TRP), 89 (hydrophobic) and 104 (2nd-CYS). These amino acids contribute to the two major features shared by the V and C-domains: The disulfide bridge (between the two cysteines 23 and 104) and the internal hydrophobic core of the domain (with the side chains of tryptophan W41 and amino acid 89). The fifth position, 118, is diverse and is characterized as being an FG loop anchor. In the IMGT system, the C-domains (C-DOMAIN and C-LIKE-DOMAIN) are delimited considering the exon delimitation, whenever appropriate, allowing the integration of strands A and G, which do not have structural alignments.

**Figure 4:**
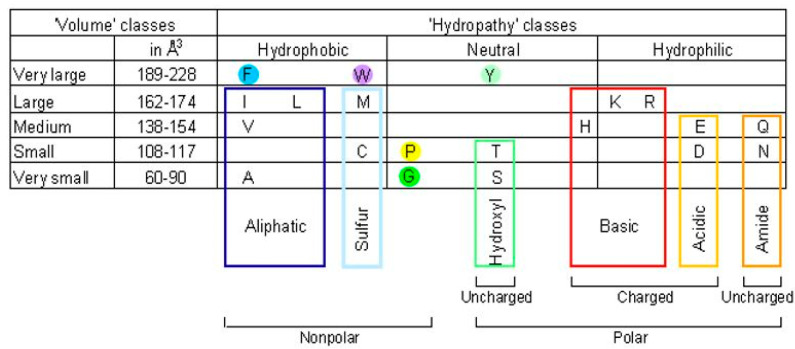
IMGT physicochemical classes of the 20 usual amino acids (AA) [52] (with permission from M-P. Lefranc and G. Lefranc, LIGM, Founders of IMGT^®^, the international ImMunoGeneTics information system^®^, https://www.imgt.org).

The 20 usual amino acids (AA) have been classified in eleven IMGT physicochemical classes [52] (IMGT^®^ https://www.imgt.org, IMGT Education > Aide-mémoire > Amino acids) (Figure 4).

## 4. IGHG, IGKC and IGLC2 Engineered Variants

One hundred and fourteen IGHG engineered variants have been defined by their IMGT gene nomenclature, the IMGT unique numbering for C-domain [68] and IMGT motifs in domain strands and/or loops (Table 4, Figure 3), with corresponding Eu positions [97] (IMGT https://www.imgt.org, IMGT Scientific chart > Correspondence between C numberings > Correspondence between the IMGT unique numbering for C-DOMAIN, the IMGT exon numbering, the EU and Kabat numberings: Human IGHG [97,98] https://www.imgt.org/IMGTScientificChart/Numbering/Hu_IGHGnber.html) (Supplementary Table S1). The IGKC and IGLC2 engineered variants involved in the structure have also been defined similarly by their IMGT gene nomenclature, the IMGT unique numbering for the C-domain [68] and IMGT motifs in the domain strands and/or loops (Table 4), with corresponding Eu positions [97] (IMGT https://www.imgt.org, IMGT Scientific chart > Correspondence between C numberings > Correspondence between the IMGT unique numbering for the C-DOMAIN, the IMGT exon numbering, the EU and Kabat numberings: Human IGKC [97,98].

**Figure 5:**
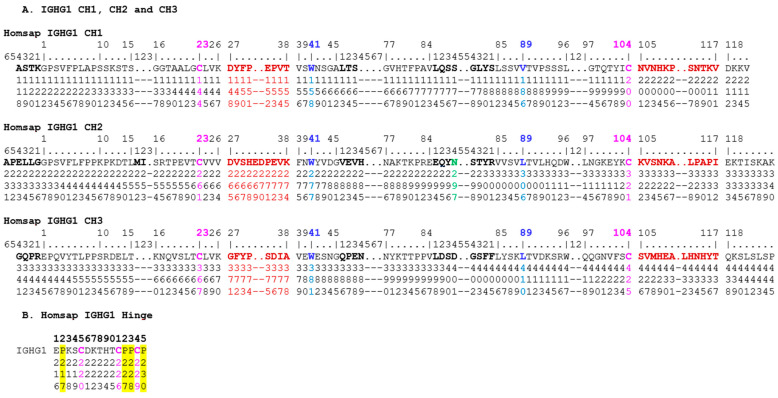
Correspondence between the *Homo sapiens* IGHG1 amino acid sequence, based on the IMGT unique numbering for the C-domain [68] and the Eu positions (shown vertically) from 118 to 445 [97]. (**A**) IGHG1 CH1, CH2 and CH3. The standardized presentation of the IMGT unique numbering on the top two lines [68] can be obtained using IMGT/DomainGapAlign [28,49,50], the IMGT reference tool for constant C-domain amino acid sequence analysis. The IMGT unique numbering for the CH1, CH2 and CH3 is shown on the first horizontal line with additional IMGT positions (by comparison to the V-domain IMGT unique numbering [67]) on line two. Amino acids at these additional positions are highlighted in bold. The Eu numbers are read vertically (on three lines top to down) at each position below the amino acid sequence. For example, the first amino acid of the Homsap IGHG1 CH1 is A1.4 (read G1, and going left, K1.1, T1.2, S1.3 and A1.4) and corresponds to Eu 118 (below A, read one top line, one second line and eight third line). The last amino acid of CH1 is a V, at position IMGT 121 (3 dots after 118), and corresponds to Eu 215 (below V, read two top line, one second line and five third line). The first amino acid of the Homsap IGHG1 CH2 A1.6 corresponds to Eu 231, whereas the last one, K, at position IMGT 125 (7 dots after 118), corresponds to Eu 340. The first amino acid of the Homsap IGHG1 CH3 G1.4 corresponds to Eu 341, whereas the last one, P, at position IMGT 125, corresponds to Eu 445. The first amino acid of the CH1, hinge, CH2 and CH3 results from the splicing. The four conserved amino acids of the C-DOMAIN C23, W41, hydrophobic 89 and C104 are highlighted in colors (C23 and C104 in pink, W41 and hydrophobic 89 (V, L) in blue). The four AA and IMGT positions C23, W41, hydrophobic 89 and C104 correspond, respectively, to Eu 144, 158, 186 and 200 in CH1, 261, 277, 306 and 321 in CH2, and 367, 381, 410 and 425 in CH3. The CH2 asparagine N84.4 of the N-glycosylation site corresponds to Eu 297 (colored in green). The amino acids of the C-domain BC-LOOP and FG-LOOP (Table 4) are highlighted in bold and brown color. (**B**) Homsap IGHG1 hinge. The hinge IMGT 1 to 15 corresponds to Eu 216 to 230. Cysteines (C) and prolines (P) with Eu positions are highlighted in pink and yellow, respectively. (Drawn by Marie-Paule Lefranc and Gérard Lefranc, LIGM, Founders and Authors of IMGT^®^, the international ImMunoGeneTics information system^®^, https://www.imgt.org, Copyright 2022.)

**Figure 6:**
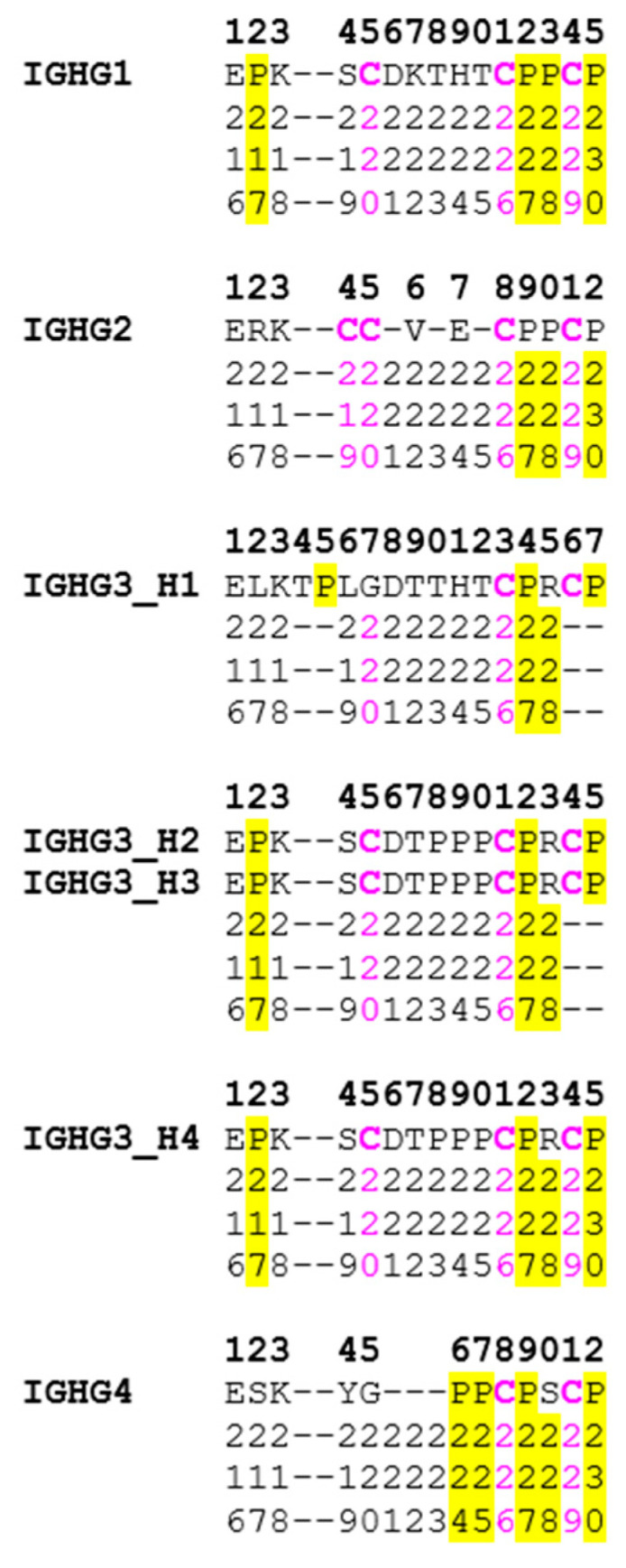
Correspondence between the *Homo sapiens* IGHG1, IGHG2, IGHG3 (4 exons) and IGHG4 IMGT numbering with the IGHG1 Eu positions. The top line indicates the IMGT numbering for the IGHG1, IGHG2 and IGHG4 hinges and for the four exons (H1 to H4) of the IGHG3 hinge. The Eu numbers are read vertically (on three lines top to down) at each position below the amino acid sequence. Dashes indicate the positions that are absent in the Eu numbering. Cysteines (C) and prolines (P) with Eu positions are highlighted in pink and yellow, respectively. (Drawn by Marie-Paule Lefranc and Gérard Lefranc, LIGM, Founders and Authors of IMGT^®^, the international ImMunoGeneTics information system^®^, https://www.imgt.org, Copyright 2022).

**Figure 7:**
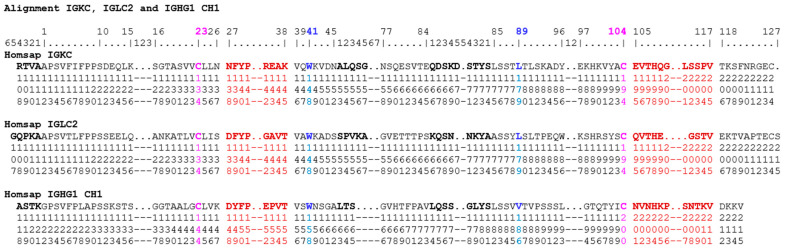
Correspondence between the *Homo sapiens* IGKC, IGLC2 and IGHG1 CH1 sequences, based on the IMGT unique numbering [68] and the Eu positions [97]. The first amino acid of each sequence results from the splicing. The IGHG1 CH1 chosen as the CH representative is from Figure 5A. The IMGT unique numbering is shown on the top horizontal line one with additional IMGT positions on line two. Amino acids at these additional positions (by comparison to the V-domain IMGT unique numbering [67]) are highlighted in bold in the Homsap IGKC, IGLC2 and IGHG1 CH1 sequences. The Eu numbers are read vertically (on three lines top to down) at each position below the amino acid sequences. For example, the first amino acid of IGKC R1.4 corresponds to Eu 108, that of IGLC2 G1.5 to Eu 107, and that of IGHG1 CH1 A1.4 to Eu 118, the last amino acid of IGKC C126 corresponds to Eu 214, that of IGLC2 S215 to ‘deduced Eu position 215′ and that of IGHG1 CH1 V at position IMGT 121 corresponds to Eu 215. The four conserved amino acids of the C-DOMAIN C23, W41, hydrophobic 89 and C104 are highlighted in colors (C23 and C104 in pink, W41 and hydrophobic 89 (L, V) in blue). The four AA and IMGT positions C23, W41, hydrophobic 89 and C104 correspond, respectively, to Eu 134, 148, 179, 194 for IGKC and IGLC2 and to Eu 144, 158, 186 and 200 in IGHG1 CH1. The amino acids of the C-domain BC-LOOP and FG-LOOP (Table 4) are highlighted in bold and brown color. (Drawn by Marie-Paule Lefranc and Gérard Lefranc, LIGM, Founders and Authors of IMGT^®^, the international ImMunoGeneTics information system^®^, https://www.imgt.org, Copyright 2022.)

The correspondence between the IMGT unique numbering and the Eu positions are provided here in a horizontal format for the IGHG1 CH1, hinge, CH2 and CH3-domains (Figure 5), and hinges of IGHG1, IGHG2, IGHG3 and IGHG4 (Figure 6), and by extension to the alignment of IGKC and IGLC2 with IGHG1 CH1 (Figure 7).

Standardized characterization has become a necessity, owing to the increasing number of engineered antibodies of effector properties [99,100] and/or various formats. Based on the IMGT Scientific chart rules, we propose a standardized IMGT nomenclature of engineered variants involved in effector properties (ADCC, ADCP and CDC), half-life and structure of therapeutical monoclonal antibodies. The standardized variant characterization comprises (1) the IMGT engineered Fc variant name (e.g. G1v1), (2) the IMGT variant definition (for each amino acid (AA) change: domain, AA in the one-letter abbreviation [52] and its position in the IMGT unique numbering for C domain [68], e.g. CH2 P1.4, (3) the IMGT amino acid changes on the IGHG CH domain with the Eu numbering between parentheses (e.g., CH2 E1.4 > P (233)), (4) the Eu numbering variant (e.g., E233P), (5) the IMGT motif positions according to the IMGT unique numbering [68], followed between parentheses, by the Eu numbering, motif with AA before and after the AA change in bold (e.g., IGHG1 CH2 1.6–3 (231–239) AP**E**LLGGPS > AP**P**LLGGPS; underlined amino acids in the motif correspond to additional positions in the IMGT unique numbering for the C-domain [68,70,71,72], e.g., APELLG and APPLLG which correspond to 1.6, 1.5, 1.4, 1.3, 1.2 and 1.1), and (6) information from the literature regarding ‘property and function’. 

These properties and functions have allowed to classify the IMGT engineered variants in 19 types (#1 to #19) corresponding to four categories. The first category ‘Effector’ refers to the variants that affect the effector properties: ADCC reduction #1 (Table 5), ADCC enhancement #2 (Table 6), ADCP and CDC enhancement #3 (Table 7), CDC enhancement #4 (Table 8), CDC reduction #5 (Table 9), ADCC and CDC reduction #6 (Table 10), B cell inhibition by the coengagement of antigen and FcγR on the same cell #7 (Table 11), knock out CH2 84.4 glycosylation #8 (Table 12), the second category ‘Half-life’ refers to the variants that affect (most of them increasing) the half-life #9 (Table 13), the third one ‘Protein A’ refers to the abrogation of binding to protein A #10 (Table 14), the fourth one ‘Structure’ refers to variants that affect the stability or structure of monospecific, bispecific or multispecific antibodies and include: formation of additional bridge stabilizing CH2 in the absence of N84.4 (297) glycosylation #11 (Table 15), prevention of IgG4 half-IG exchange #12 (Table 16), hexamerisation #13 (Table 17), knobs-into-holes and the enhancement of heteropairing H-H of bispecific antibodies #14 (Table 18), suppression of inter H-L and/or inter H-H disulfide bridges #15 (Table 19), site-specific drug attachment #16 (Table 20), enhancement of hetero pairing H-L of bispecific antibodies #17 (Table 21), control of half-IG exchange of bispecific IgG4 #18 (Table 22), reducing acid-induced aggregation #19 (Table 23).

In the tables, the different columns correspond to the items of the standardized variant characterization detailed above. Engineered amino acid changes are in bold in the IMGT variants (red before the change, green after the change. The motif is in yellow and shown before and after the AA change(s).

The variants involved in antibody-dependent cellular cytotoxicity (ADCC) reduction. include nine *Homo sapiens* IGHG1 variants, which comprise: G1v1 [1], G1v2 [1], G1v3 [1], G1v5 [6], G1v47 [37], G1v50 (the variant G1v50 is a variant combining the G1v1, G1v2, G1v3 and G1v47 amino acid changes), G1v52 ‘GRLR’, G1v66 and G1v67 (Table 5).

The variants involved in antibody-dependent cellular cytotoxicity (ADCC) enhancement include nine variants, of which six *Homo sapiens* IGHG1 variants: G1v6 [3], G1v7 ‘DE’ [4], G1v8 ‘DLE’ ‘3M’ [4] [25], G1v9 [14], G1v10 [15] and G1v11 [15]; one *Homo sapiens* IGHG2 variant: G2v1 [1]; one *Homo sapiens* IGHG4 variant: G4v1 [1]; and one *Mus musculus* IGHG2B variant: Musmus G2Bv1 [5] (Table 6).

The variants involved in antibody-dependent cellular cytotoxicity (ADCC) and antibody-dependent cellular phagocytosis (ADCP) enhancement include three *Homo sapiens* IGHG1 variants: G1v12 ‘GASDALIE’ [26], G1v13 ‘GASDIE’ ‘ADE’ [16] and G1v45 ‘GAALIE’ (Table 7).

The variants involved in complement-dependent cytotoxicity (CDC) enhancement include 8 variants, of which seven *Homo sapiens* IGHG1 variants: G1v5 [6], G1v15 [6], G1v16 [6], G1v17 ‘EFT’ [18], G1v18 [19], G1v35 ‘SE’ [18,27] and the chimeric G1G3v1 [17], and one IGHG4 variant: G4v2 [8] (Table 8).

The variants involved in complement-dependent cytotoxicity (CDC) reduction include six variants, of which three *Homo sapiens* IGHG1 variants: G1v8 ‘DLE’ [4], G1v19 [2] and G1v20 [2,39]; and three *Mus musculus* IGHG2B variants: Musmus G2Bv2 [7], Musmus G2Bv3 [7] and Musmus G2Bv4 [7] (Table 9).

The variants involved in antibody-dependent cellular cytotoxicity (ADCC) and complement-dependent cytotoxicity (CDC) reduction include 32 variants and four variant associations, of which 22 *Homo sapiens* IGHG1 variants: G1v4 [2], G1v14 ‘LALA’ [21,39], G1v14-1, G1v14-4, G1v14-48, G1v14-49 ‘LALAPG’ [40], G1v14-67, G1v23 [20], G1v38 [35], G1v39 ‘FES’ ‘TM’ [20,24], G1v40, G1v41 [20,24], G1v43, G1v48, G1v49 [40], G1v51, G1v53 ‘FQQ’, G1v59 [41], G1v60, G1v63, G1v65, G1v70 and one association G1v53-G1v21 ‘FQQ-YTE’ [38]; three *Homo sapiens* IGHG2 variants: G2v2 ‘IgG2m4′ [23], G2v3 ‘G2sigma’ [24] and the chimeric G2G4v1 [22]; five *Homo sapiens* IGHG4 variants: G4v3 ‘LE’ [20], G4v3-49 ‘LEPG’ [40], G4v4 ‘FALA’ [21], G4v7, G4v49 [40] and three associations G4v3-G4v5 ‘SPLE’ [12,20], G4v3-49-G4v5 ‘SPLEPG’ [40] [12] and G4v4-G4v5 ‘IgG4ProAlaAla’ [12,24] and two *Canis lupus familiaris* IGHG2 variants: CanlupfamG2v1 and CanlupfamG2v2 (Table 10).

The variants involved in B cell inhibition by coengagement of antigen and FcγR on the same cell include one *Homo sapiens* IGHG1 variant: G1v25 [33,34] (Table 11).

The variants involved in knock out CH2 84.4 glycosylation include five variants, of which three *Homo sapiens* IGHG1 variants: G1v29 [42], G1v30 [42], G1v36; one *Homo sapiens* IGHG4 variant: G4v36; and one *Canis lupus familiaris* IGHG2 variant: Canlupfam G2v29 (Table 12).

The variants involved in half-life increase or decrease include 13 variants, 12 of them increase half-life, of which five *Homo sapiens* IGHG1 variants: G1v21 ‘YTE’ [9,29,30,31,32], G1v22 [30], G1v24 [32], G1v42 [30] and G1v46; 3 *Homo sapiens* IGHG2 variants: G2v4 [10], G2v5 [10] and G2v6 [10]; one *Homo sapiens* IGHG3 variant: G3v1 [11]; three *Homo sapiens* IGHG4 variants: G4v21 ‘YTE’ [30], G4v22 [36] and G4v24. One variant G2v8-1 abrogates binding to FCGRT (FcRn) (Table 13).

The variants involved in abrogation of binding to Protein A include one *Homo sapiens* IGHG4 variant: G4v8 (Table 14).

The variants involved in formation of additional bridge stabilizing CH2 in the absence of N84.4 (Eu 297) glycosylation include four *Homo sapiens* IGHG1 variants: G1v54, G1v54-29, G1v54-30 and G1v54-36 (Table 15).

The variants involved in prevention of IgG4 half-IG exchange include two *Homo sapiens* IGHG4 variants: G4v5 [12] and G4v6 [13] (Table 16).

The variants involved in hexamerisation include one *Homo sapiens* IGHG1 variant: G1v34 (Table 17).

The variants involved in knobs-into-holes and enhancement of heteropairing H-H of bispecific antibodies include six *Homo sapiens* IGHG1 variants: G1v26 knob [28] and G1v31 hole [28], G1v32 knob and G1v33 hole, G1v68 and G1v69 (Table 18).

The variants involved in suppression of inter H-L and/or inter H-H disulfide bridges includes three *Homo sapiens* IGHG1 variants: G1v37, G1v61 and G1v62 (Table 19).

The variants involved in site-specific drug attachment include six *Homo sapiens* IGHG1 variants: G1v27, G1v28, G1v44, G1v55, G1v56 and G1v64 (Table 20).

The variants involved in enhancement of hetero pairing H-Linclude two *Homo sapiens* IGHG1 variants: G1v57 used in association with *Homo sapiens* IGKC variant: KCv57, and G1v58, used in association with *Homo sapiens* IGLC2 variant: LC2v58 (Table 21).

The variants involved in control of half-IG exchange of bispecific IgG4 antibodies include one *Homo sapiens* IGHG4 variant: G4v10 (Table 22).

The variants involved in reducing acid-induced aggregation include one *Homo sapiens* IGHG2 variant: G2v7 (Table 23).

Two variants have been assigned to two properties belonging to different types and are therefore found in two tables, G1v5 (Table 5 and Table 8) and G1v8 (Table 6 and Table 9).

Supplementary Table S2 provides the variants of Table 5, Table 6, Table 7, Table 8, Table 9, Table 10, Table 11, Table 12, Table 13, Table 14, Table 15, Table 16, Table 17, Table 18, Table 19, Table 20, Table 21, Table 22 and Table 23 in an alphanumeric order of the IMGT engineered variants involved in the effector properties (ADCC, ADCP and CDC), half-life and structure of the therapeutical monoclonal antibodies.

## 5. Conclusions

The therapeutic monoclonal antibody engineering field is the most promising in the medical field. A standardized analysis of IG genomic and expressed sequences, structures and interactions is crucial for a better molecular understanding and comparison of the mAb specificity, affinity, half-life, Fc effector properties and potential immunogenicity. IMGT has provided the concepts for the IG loci description of newly sequenced genomes [2], antibody structure/function characterization [4], antibody engineering (single chain Fragment variable (scFv), phage displays, combinatorial libraries) and antibody humanization (chimeric, humanized and human antibodies). IMGT^®^ standardization allows the repertoire analysis and antibody humanization studies to move to novel, high-throughput methodologies with the same high-quality criteria. The CDR-IMGT lengths are now required for mAb INN applications and are included in the WHO-INN definitions (84–86). The characterization of the IGHG engineered variants for effector properties, half-life increase, and new structures of bi- and multi-specific antibodies brings a new level of standardized information in the comparative analysis of therapeutic antibodies.

## Figures and Tables

**Table 1:** The IMGT databases, tools and web resources (‘The IMGT Marie-Paule Page’) for sequences, genes and structures.

	IMGT Databases	IMGT Tools	IMGT Web Resources ‘The IMGT Marie-Paule Page’
Sequences	IMGT/LIGM-DB [24,25] IMGT/PRIMER-DB IMGT/CLL-DB	IMGT/V-QUEST [31,32,33,34,35,36] IMGT/JunctionAnalysis [37,38] IMGT/Automat [39,40] IMGT/HighV-QUEST [36,41,42,43,44,45] IMGT/StatClonotype [46,47] IMGT/PhyloGene IMGT/Allele-Align	Standardized keywords and labels [53,54] Standardized labels [55,56,57,58] IMGT Repertoire (IG and TR, MH, RPI Alignments of alleles Protein displays Tables of alleles CDR-IMGT lengths Allotypes [59,60] Isotypes, etc.
Genes	IMGT/GENE-DB [26]	IMGT/LIGMotif [48] IMGT/LocusView IMGT/GeneView IMGT/GeneSearch IMGT/CloneSearch IMGT/GeneInfo	Gene and allele nomenclature [1,2,3,4,5,7,8,9,10,61,62,63] Chromosomal localizations Locus representations Locus description Gene exon/intron splicing sites Gene tables Potential germline repertoires Lists of genes Correspondence between nomenclatures.
Structures	IMGT/2Dstructure-DB IMGT/3Dstructure-DB [27,28,29] IMGT/mAb-DB [30]	IMGT/DomainGapAlign [28,49,50] IMGT/DomainDisplay IMGT/StructuralQuery IMGT/Collier-de-Perles [51]	IMGT unique numbering per domain [64,65,66,67,68,69,70,71,72] 2D Colliers de Perles (IG and TR, MH, RPI) [51,73,74,75,76,77] IMGT classes for amino acid physicochemical properties [52] IMGT Colliers de Perles reference profiles [52] 3D representations.

**Table 2:** IMGT-ONTOLOGY axioms, concepts and IMGT Scientific chart rules.

IMGT-ONTOLOGY Axioms and Concepts		IMGT Scientific Chart Rules
IDENTIFICATION [54]	Concepts of identification [53]	Standardized keywords [53,54] (e.g., clonotype, paratope, epitope, variant, Fc receptor, FcR) (1).
DESCRIPTION [56]	Concepts of description [55]	Standardized labels and annotations [55,56,57,58] (e.g., CDR-IMGT [57], FR-IMGT [58], antibody description [84])
CLASSIFICATION [63]	Concepts of classification [62]	Reference sequences Standardized IG and TR gene nomenclature (group, subgroup, gene, allele) [1,2,3,4,5,7,8,9,10,61,62,63] (1).
NUMEROTATION [64]	Concepts of numerotation [65,66,67,68,69,70,71,72]	IMGT unique numbering for V- and V-LIKE domains [65,66,67] C- and C-LIKE domains [68] G- and G-LIKE domains [69] IMGT Colliers de Perles [73,74,75,76,77]
ORIENTATION	Concepts of orientation	Chromosome orientation Locus orientation Gene orientation DNA strand orientation Domain beta-strand orientation
OBTENTION	Standardized origin Standardized methodology	

Keyword use versus gene name nomenclature for defining a receptor: in this paper, this concerns the related proteins of immune interest (RPI) such as the Fc receptor’s gamma. Owing to the diversity and multiplicity of these receptors, and in the absence of standardized sequence characterization in functional analysis, these receptors are usually identified with keywords, for example for *Homo sapiens*, FcγR, FcγRI, FcγRII, FcγRIII and so on. However, it should be noted that, when there is no ambiguity as to the interactive chain involved, the HGNC gene name should be used (FCGR1A, FCGR2A, FCGR2B, FCRG2C, FCGR3A and FCGR3B). This rule is applied in this paper for the neonatal Fc receptor (FcRn), which is made of the interactive Fc gamma receptor and transporter (FCGRT) chain that is associated with B2M.

**Table 3:** The immunoglobulin IgG1 receptor, chain and domain structure labels and correspondence with sequence labels. IMGT standardized labels are in capital letters. They are shown with the example *Homo sapiens* IgG1-kappa.

IG Structure Labels (IMGT/3Dstructure-DB [27,28,29])				Sequence Labels (IMGT/LIGM-DB [24,25])
Receptor	Chain	Domain Type	Domain	Region ^1^
IG-GAMMA-1_KAPPA	H-GAMMA-1	V	VH	V-D-J-REGION
		C	CH1	C-REGION ^2^
		C	CH2	
		C	CH3	
	L-KAPPA	V	V-KAPPA	V-J-REGION
		C	C-KAPPA	C-REGION

**^1.^** The VH-domain (or V-D-J-REGION) and the VL-domain (V-KAPPA or V-LAMBDA) (or V-J-REGION) are encoded by rearranged V-(D)-J genes, whereas the remainder of the chain is the C-REGION (encoded by a C gene). The C-REGION comprises one C-domain (C-KAPPA or C-LAMBDA) for the L chain, or several C-domains (CH) for the H chain. ^2^ The heavy chain C-REGION also includes the HINGE-REGION, and for membrane IG (mIG), the CONNECTING-REGION (CO), TRANSMEMBRANE-REGION (TM) and CYTOPLASMIC-REGION (CY); for secreted IG (sIG), the C-REGION includes CHS instead of CO, TM and CY.

**Table 4:** C-domain strands, turns and loops, IMGT positions and lengths, based on the IMGT unique numbering for C-domain (C-DOMAIN and C-LIKE-DOMAIN) [68]. (With permission from M-P. Lefranc and G. Lefranc, LIGM, Founders of IMGT^®^, the international ImMunoGeneTics information system^®^, https://www.imgt.org).

C Domain Strands, Turns and Loops ^a^	IMGT Position ^b^	Lengths ^c^	Characteristic IMGT Residue@Position ^d^
A-STRAND	1–c15	15 (14 if gap at 10)	
AB-TURN	15.1–15.3	0-3	
B-STRAND	16–26	11	1st-CYS 23
BC-LOOP	27–31 34–38	10 (or less)	
C-STRAND	39–45	7	CONSERVED-TRP 41
CD-STRAND	45.1–45.9	0–9	
D-STRAND	77–84	8 (or 7 if gap at 82)	
DE-TURN	84.1–84.7 85.1–85.7	0–14	
E-STRAND	85–96	12	hydrophobic 89
EF-TURN	96.1–96.2	0–2	
F-STRAND	97–104	8	2nd-CYS 104
FG-LOOP	105–117	13 (or less, or more)	
G-STRAND	118–128	11 (or less)	

^a^ IMGT labels (concepts of description) are written in capital letters (no plural) [55,56]. ^b^ based on the IMGT unique numbering for C-domain (C-DOMAIN and C-LIKE-DOMAIN) [68]. ^c^ in number of amino acids (or codons). ^d^ IMGT Residue@Position is a given residue (usually an amino acid) or a given conserved property amino acid class, at a given position in a domain, based on the IMGT unique numbering [68].

**Table 5:** IMGT nomenclature, Eu positions and IMGT motif of engineered Fc variants involved in antibody-dependent cellular cytotoxicity (ADCC) reduction (Effector #1).

IMGT Engineered Fc Variant Name	IMGT Engineered Variant Definition	IMGT Amino Acid Changes on IGHG CH Domain (Eu Numbering between Parentheses)	Amino Acid Changes With the Eu Positions	Motif Identifiable in Gene and Domain with Positions According to the IMGT Unique Numbering and with Eu Positions between Parentheses	1. Property and Function	2. Property and Function
**G1v1**	**CH2** **P1.4**	CH2 **E**1.4 > **P** (233)	E233P	IGHG1 CH2 1.6–3 (231–239) AP**E**LLGGPS > AP**P**LLGGPS	**ADCC reduction.** Prevents FcγRI binding [101]	
**G1v2**	**CH2** **V1.3**	CH2 **L**1.3 > **V** (234)	L234V	IGHG1 CH2 1.6–3 (231–239) APE**L**LGGPS > APE**V**LGGPS	**ADCC reduction** Decreases FcγRI binding [101]	
**G1v3**	**CH2** **A1.2**	CH2 **L**1.2 > **A** (235)	L235A	IGHG1 CH2 1.6–3 (231–239) APEL**L**GGPS > APEL**A**GGPS	**ADCC reduction.** Prevents FcγRI binding [101]	
**G1v5**	**CH2** **W109**	**CH2** **K**109 > **W** (326)	K326W	IGHG1 CH2 FG 105–117 (322–332) KVSN**K**A..LPAPI > KVSN**W**A..LPAPI	**ADCC reduction** [102]	**CDC enhancement.** Increases C1q binding [102]
**G1v47**	**CH2** **delG1.1**	CH2 **G**1.1 > del (326)	G236del	IGHG1 CH2 1.6–3 (231–239) APELL**G**GPS > APELLGPS	**ADCC reduction.** Eliminates binding to FcγRI, FcγRIIA, FcγRIIIA [103]	
**G1v50**	**CH2** **P1.4** **V1.3** **A1.2** **delG1.1**	CH2 **E**1.4 > **P** (233), **L**1.3 > **V** (234), **L**1.2 > **A** (235), **G**1.1 > del (236)	E233P, L234V, L235A, G236del	IGHG1 CH2 1.6–3 (231–239) AP**ELLG**GPS > AP**PVA-**GPS	**ADCC reduction.** Decreases FcgammaR binding (G2-like motif). [Combines G1v1, v2, v3 and v47]	
**G1v52**	**CH2** **R1.1,** **R113**	CH2 **G**1.1 > **R** (231) **L**113 > **R** (328)	G236R, L328R GRLR	IGHG1 CH2 1.6–3 (231–239) APELL**G**GPS > APELL**R**GPS IGHG1 CH2 FG 105–117 (322–332) KVSNKA..**L**PAPI > KVSNKA..**R**PAPI	**ADCC reduction.** Abrogates FcgammaR binding	
**G1v66**	**CH2** **A27**	CH2 **D**27 > **A**	D265A	IGHG1 CH2 23–31 (261–269) CVVV**D**VSHE > CVVV**A**VSHE	**ADCC reduction.** Reduces FcγR binding.	
**G1v67**	**CH2** **S27**	CH2 **D**27 > **S**	D265S	IGHG1 CH2 23–31 (261–269) CVVV**D**VSHE > CVVV**S**VSHE	**ADCC reduction.** Reduces FcγR binding.	

Engineered amino acid changes are in bold in the IMGT variants (red before the change, green after the change. The motif is in yellow and shown before and after the AA change(s). Amino acids of the motifs at additional positions in the IMGT unique numbering for C-domain [68] (by comparison to the V-domain IMGT unique numbering [67]) are underlined. Alias variant names found in the literature are written in blue in column 4 ‘Amino Acid Changes with the Eu Positions’. The background color indicates a reduction (pink color) or an enhancement (green color) of the involved effector ‘Property and Function’. For other ‘Property and Function’, background colors refer to structure (yellow), half-life (pale blue color) or protein A (pale orange).

**Table 6:** IMGT nomenclature, Eu positions and IMGT motif of engineered Fc variants involved in antibody-dependent cellular cytotoxicity (ADCC) enhancement (Effector #2).

IMGT Engineered Fc Variant Name	IMGT Engineered Variant Definition	IMGT Amino Acid Changes on IGHG CH Domain (Eu Numbering between Parentheses)	Amino Acid Changes with the Eu Positions	Motif Identifiable in Gene and Domain with Positions According to the IMGT Unique Numbering and with Eu Positions between Parentheses	1. Property and Function	2. Property and Function	3D
**G1v6**	**CH2** **A85.4, ** **A118, ** **A119**	CH2 **S**85.4 > **A**(298), **E**118 > **A** (333), **K**119 > **A** (334)	S298A, E333A, K334A	IGHG1 CH2 84.1–85.1 (294–301) EQYN**S**TYR > EQYN**A**TYR FG 105–117,118,119 (322–334) KVSNKA..LPAPI****EK** > ** KVSNKA..LPAPI****AA****	**ADCC enhancement.** Increases FcγRIIIa binding [104]		
**G1v7**	**CH2** **D3, ** **E117**	CH2 **S**3 > **D** (239), **I**117 > **E** (332)	S239D, I332E DE	IGHG1 CH2 1.6–3 (231–239) APE**LL**GGP**S** > APE**LL**GGP**D** FG 105–117 (322–332) KVSNKA..LPAP****I** > ** KVSNKA..LPA**P******E****	**ADCC enhancement.** Increases FcγRIIIA binding [105]		
**G1v8**	**CH2** **D3, ** **L115, ** **E117**	CH2 **S**3> **D** (239), **A**115 > **L** (330), **I**117 > **E** (332)	S239D, A330L, I332E DLE, 3M	IGHG1 CH2 1.6–3 (231–239) APELLGGP**S** > APELLGGP**D** FG 105–117 (322–332) KVSNKA..LP****A****P****I** > ** KVSNKA..LP****L****P****E****	**ADCC enhancement.** Increases FcRIIIA binding [105]	Decreases FcγRIIB binding [105]	3D [106]
**G1v9**	**CH2** **L7, ** **P83, ** **L85.2, ** **I88**. **CH3** **L83**	CH2 **F**7 > **L** (243), **R**83 > **P** (292), **Y**85.2 > **L **(300), **V**88 > **I **(305) CH3 **P**83 > **L **(396)	F243L, R292P, Y300L, V305I, P396l LPLIL	IGHG1 CH2 6–10 (242–246) L**F**PPK > L**L**PPK 83–88 (292–305) **R**EEQYNS**T****Y**RVVS**V** > **P**EEQYNST**L**RVVS**I** CH3 83–84.4 (396–401) **P**VLDSD > **I**VLDSD	**ADCC enhancement.** 100% increase. [107]		
**G1v10**	**CH2** **Y1.3, ** **Q1.2, ** **W1.1, ** **M3, ** **D30, ** **E34, ** **A85.4**	CH2 **L**1.3 > **Y** (234), **L**1.2 > **Q** (235), **G**1.1 > **W** (236), **S**3 > **M** (239), **H**30 > **D** (268), **D**34 > **E** (270), **S**85.4 > **A** (298)	L234Y, L235Q, G236W, S239M, H268D, D270E, S298A	IGHG1 CH2 1.6–3 (231–239) APE**LLG**GP**S** > APE**YQW**GP**M** 27–31,34 (265–270) DVS**H**E**D** > DVS**D****E****E** 84.1–85.1 (294–301) EQYN**S**TYR > EQYN**A**TYR	**ADCC enhancement.** Increases FcγIIIA binding [108] >2000-fold (F158), >1000-fold (V158) in the association of G1v10 and G1v11 [108]		
**G1v11**	**CH2** **E34**, **D109**, **M115**, **E119**	CH2 **D**34 > **E** (270), **K**109 > **D** (326), **A**115 > **M** (330) **K**119 > **E** (334)	D270E, K326D, A330M, K334E	IGHG1 CH2 27–31,34 (265–270) DVSHE**D** > DVSHE**E** FG 105–117,118,119 (322–334) KVSN**K**A..LP****A****PIE****K** >** KVSN****D****A..LP****M****PIE****E****	**ADCC enhancement.** Increases FcγIIIA binding [108] >2000-fold (F158), >1000-fold (V158) in the association of G1v10 and G1v11 [108]		
**G2v1**	**CH2** **L1.3, ** **L1.2, ** **G1.1**	CH2 **V**1.2 > **LL**(234,235) **A**1.1 > **G**(236)	V235LL, A236G	IGHG2 CH2 1.6–3 (231–239) AP.P**VA****G**PS > APP**LL****G**GPS	**ADCC enhancement.** Confers FcγRI binding (WT does not show any binding capacity) [101]		
**G4v1**	**CH2** **L1.3**	CH2 **F**1.3 > **L** (234)	F234L	IGHG4 CH2 1.6–3 (231–239) APE**F**LGGPS > APE**L**LGGPS	**ADCC enhancement.** Increases FcγRI affinity [101]		
***Mus musculus*** **G2Bv1**	**CH2** **L1.2**	CH2 **E**1.2 > **L** (235)	E235L	IGHG2B CH2 1.6–3 (231–239) APNL**E**GGPS > APNL**L**GGPS	**ADCC enhancement.** Increases FcγRI affinity [109]		

**Table 7:** IMGT nomenclature, Eu positions and IMGT motif of engineered Fc variants involved in antibody-dependent cellular cytotoxicity (ADCC) and antibody-dependent cellular phagocytosis (ADCP) enhancement (Effector #3).

IMGT Engineered Fc Variant Name	IMGT Engineered Variant Definition	IMGT Amino Acid Changes on IGHG CH Domain (Eu Numbering between Parentheses)	Amino Acid Changes with the Eu Positions	Motif Identifiable in Gene and Domain with Positions According to the IMGT Unique Numbering and with Eu Positions between Parentheses	1. Property and Function	2. Property and Function	3D
**G1v12**	**CH2** **A1.1, ** **D3, ** **L115, ** **E117 **	CH2 **G**1.1 > **A** (236), **S**3 > **D** (239), **A**115 > **L** (330), **I**117 > **E** (332)	G236A, S239D, A330L, I332E GASDALIE	IGHG1 CH2 1.6–3 (231–239) APELL**G**GP**S** > APELL**A**GP**D** FG 105–117 (322–332) KVSNKA..LP****A****P****I** > ** KVSNKA..LP****L****P****E****	**ADCC enhancement.** Increases FcγRIIIA binding [110]	**ADCP enhancement.** NK cell activation. Increases FcγRIIA binding [110]	5d4q, 5d6d
**G1v13**	**CH2** **A1.1,** **D3,** **E117**	CH2 **G**1.1 > **A** (236), **S**3 > **D** (239), **I**117 > **E** (332)	G236A, S239D, I332E GASDIE, ADE	IGHG1 CH2 1.6–3 (231–239) APELL**G**GP**S** > APELL**A**GP**D** FG 105–117 (322–332) KVSNKA..LPAP****I** > ** KVSNKA..LPAP****E****	**ADCC enhancement.** Increases FcγIIIA binding [111]	**ADCP enhancement.** NK cell activation. Increases FcγRIIA binding (70>fold)Increases FcγRIIA/FcγRIIB binding ratio (15-fold) [111]	
**G1v45**	**CH2** **A1.1, ** **L115, ** **E117**	CH2 **G**1.1 > **A** (236), **A**115 > **L** (330), **I**117 > **E**(332)	G236A, A330L, I332E GAALIE	IGHG1 CH2 1.6–3 (231–239) APELL**G**GPS > APELL**A**GPS FG 105–117 (322–332) KVSNKA..LP****A****P****I** >** KVSNKA..LP****L****P****E****	**ADCC enhancement** Increases FcγIIIA binding	**ADCP enhancement** NK cell activation	

**Table 8:** IMGT nomenclature, Eu positions and IMGT motif of engineered Fc variants involved in complement-dependent cytotoxicity (CDC) enhancement (Effector #4).

IMGT Engineered Variant Name	IMGT Engineered Variant Definition	IMGT Amino Acid Changes on IGHG CH Domain (Eu Numbering between Parentheses)	Amino Acid Changes with the Eu Positions	Motif Identifiable in Gene and Domain with Positions According to the IMGT Unique Numbering and with Eu Positions between Parentheses	1. Property and Function	2. Property and Function
**G1v5**	**CH2** **W109**	**CH2** **K**109 > **W** (326)	K326W	IGHG1 CH2 FG 105–117 (322–332) KVSN**K**A..LPAPI > KVSN**W**A..LPAPI	**CDC enhancement.** Increases C1q binding [102]	**ADCC reduction** [102].
**G1v15**	**CH2** **S118**	CH2 **E**118 > **S** (333)	E333S	IGHG1 CH2 FG 105–117,118 (322–333) KVSNKA..LPAPI**E** > KVSNKA..LPAPI**S**	**CDC enhancement.** Increases C1q binding [102]	
**G1v16**	**CH2** **W109**, **S118**	CH2 **K**109 > **W** (326), **E**118 > **S** (333)	K326W, E333S	IGHG1 CH2 FG 105–117,118 (322–333) KVSN**K**A..LPAPI**E** > KVSN**W**A..LPAPI**S**	**CDC enhancement.** Increases C1q binding [102]	
**G1v17**	**CH2** **E29**, **F30**, **T107**	CH2 **S**29 > **E** (267), **H**30 > **F**(268), **S1**07 > **T** (324)	S267E, H268F, S324T EFT	IGHG1 CH2 27–31 (265–269) DV**SH**E > DV**EF**E FG 105–117 (322–332) KV****S****NKA..LPAP**I >** KV****T****NKA..LPAPI	**CDC enhancement** Increases C1q binding [112]	
**G1v18**	**CH3** **R1**, **G109**, **Y120**	CH3 **E**1 > **R** (345), **E**109 > **G** (430), **S**120 > **Y** (440)	E345R, E430G, S440Y	IGHG1 CH3 1.4–2 (341–346) GQPR**E**P > GQPR**R**P 105–110 (426–431) SVMH**E**A > SVMH**G**A 118–125 (438–445) QK**S**LSLSP > QK**Y**LSLSP	**CDC enhancement.** Increases C1q binding [113]. The triple mutant IgG1-005-RGY (IGHG1v18) form IgG1 hexamers [113]	Favors IgG1 hexamerization.
**G1v35**	**CH2** **E29**	CH2 **S**29 > **E** (267)	S267E SE	IGHG1 CH2 27–31 (265–269) DV**S**HE > DV**E**HE	**CDC enhancement.** Increases C1q binding [112]	Binds to FCGRT and FcγRIIB, but not to other FcγR in a mouse model [114].
**G1G3v1**	**CH2** **Q38**, **K40**, **F85.2**	CH2 **K**38 > **Q** (274), **N**40 > **K** (276), **Y**85.2 > **F** (300)	K274Q, N276K, Y300F chimere G1–G3 (1)	IGHG1 CH2 34–41 (270–277) DPEV**K**F**N****W** > DPEV**Q**F**K**W 84.1–85.1 (294–301) EQYNST**Y**R > EQYNST**F**R	**CDC enhancement.** Increases C1q binding [115].	
**G4v2**	**CH2** **P116**	CH2 **S**116 > **P**(331)	S331P	IGHG4 CH2 FG 105–117 (322–332) KVSNKG..LPS**S**I > KVSNKG..LPS**P**I	**CDC enhancement **[116]. (G1-, G2-, G3-like).	

(1) The chimeric chain is the IGHG1*01 CH1-hinge—IGHG3*01 CH2-CH3. Amino acids Q38, K40 (CH2) and F85.2 (CH3) are from IGHG3*01. The changes are shown in comparison to the IGHG1*01 amino acids at the same positions as K38, N40 (CH2) and Y85.2 (CH3).

**Table 9:** IMGT nomenclature, Eu positions and IMGT motif of engineered Fc variants involved in complement-dependent cytotoxicity (CDC) reduction (Effector #5].

IMGT Engineered Variant Name	IMGT Engineered Variant Definition	IMGT Amino Acid Changes on IGHG CH Domain (Eu Numbering between Parentheses)	Amino Acid Changes with the Eu Positions	Motif Identifiable in Gene and Domain with Positions According to the IMGT Unique Numbering and with Eu Positions between Parentheses	Property and Function
**G1v8**	**CH2** **D3, ** **L115, ** **E117**	CH2 **S**3 > **D** (239), **A**115 > **L** (330), **I**117 > **E** (332)	S239D, A330L, I332E DLE	IGHG1 CH2 1.6–3 (231–239) APELLGGP**S** > APELLGGP**D** FG 105–117 (322–332) KVSNKA..LP****A****P****I** > ** KVSNKA..LP****L****P****E****	**CDC reduction.** Ablates CDC [105]
**G1v19**	**CH2** **A34**	CH2 **D**34 > **A** (270)	D270A	IGHG1 CH2 34–41 (270–277) **D**PEVKFNW > **A**PEVKFNW	**CDC reduction.** Reduces C1q binding [117]
**G1v20**	**CH2** **A105**	CH2 **K**105 > A (322)	K322A	IGHG1 CH2 FG 105–117 (322–332) ****K****VSNKA..LPAP****I** >** ****A****VSNKA..LPAPI	**CDC reduction.** Reduces C1q binding [117,118]
***Mus musculus* G2Bv2**	**CH2** **A101**	CH2 **E**101 > **A**(318)	E318A (2)	IGHG2B CH2 100–110 K**E**FKCKVNNKD > K**A**FKCKVNNKD	**CDC reduction.** Reduces C1q binding [119]
***Mus musculus* G2Bv3**	**CH2** **A103**	CH2 **K**103 > **A**(320)	K320A (2)	IGHG2B CH2 100–110 KEF**K**CKVNNKD > KEF**A**CKVNNKD	**CDC reduction.** Reduces C1q binding [119]
***Mus musculus* G2Bv4**	**CH2** **A105**	CH2 **K**105 > **A**(322)	K322A (2)	IGHG2B CH2 100–110 KEFKC**K**VNNKD > KEFKC**A**VNNKD	**CDC reduction.** Reduces C1q binding [119]

(2) *Mus musculus* IGHG2B CH2 E101, K103 and K105 form a common core in the interactions of IgG and C1q [119].

**Table 10:** IMGT nomenclature, Eu positions and IMGT motif of engineered Fc variants involved in antibody-dependent cellular cytotoxicity (ADCC) and complement-dependent cytotoxicity (CDC) reduction (Effector #6).

IMGT Engineered Fc Variant Name	IMGT Engineered Variant Definition	IMGT Amino Acid Changes on IGHG CH Domain (Eu Numbering between Parentheses)	Amino Acid Changes with the Eu Positions	Motif Identifiable in Gene and Domain with Positions According to the IMGT Unique Numbering and with Eu Positions between Parentheses	1. Property and Function	2. Property and Function	3. 3D and Property and Function
**G1v4**	CH2 **A114**	**CH2** **P**114 > **A** (329)	P329A)	IGHG1 CH2 FG 105–117 (322–332) KVSNKA..L**P**API > KVSNKA..L**A**API	**ADCC reduction**. Reduces FcγR binding [117]	**CDC reduction**. Reduces C1q binding [117]	
**G1v14**	**CH2** **A1.3, ** **A1.2**	CH2 **L**1.3 > **A** (234), **L**1.2 > A (235)	L234A, L235A LALA	IGHG1 CH2 1.6–3 (231–239) APE**LL**GGPS > APE**AA**GGPS	**ADCC reduction**. Reduces FcγR binding [118,120]	**CDC reduction**. Reduces C1q binding [118,120]	
**G1v14-1**	**CH2** **A1.3,** **A1.2,** **A1**	CH2 **L**1.3 > **A** (234), **L**1.2 > **A** (235), **G**1 > **A** (237)	L234A, L235A, G237A	IGHG1 CH2 1.6–3 (231–239) APE**LL**G**G**PS > APE**AA**G**A**PS	**ADCC reduction**. Reduces FcγR binding.	**CDC reduction**. Reduces C1q binding.	
**G1v14-4**	**CH2** **A1.3, ** **A1.2,** **A114**	CH2 **L**1.3 > **A** (234), **L**1.2 > **A** (235), **P**114 > **A** (329)	L234A, L235A, P329A	IGHG1 CH2 1.6–3 (231–239) APE**LL**GGPS > APE**AA**GGPS FG 105–117 (322–332) KVSNKA..L**P**API > KVSNKA..L**A**API	**ADCC reduction**. Reduces FcγR binding.	**CDC reduction**. Reduces C1q binding.	
**G1v14-48**	**CH2** **A1.3, ** **A1.2,** **R113 **	CH2 **L**1.3 > **A** (234), **L**1.2 > **A** (235), **L**113 > **R** (328)	L234A, L235A, L328R	IGHG1 CH2 1.6–3 (231–239) APE**LL**GGPS > APE**AA**GGPS FG 105–117 (322–332) KVSNKA..**L**PAPI > KVSNKA..**R**PAPI	**ADCC reduction**. Reduces FcγR binding.	**CDC reduction**. Reduces C1q binding.	
**G1v14-49**	**CH2** **A1.3, ** **A1.2, ** **G114**	CH2 **L**1.3 > **A** (234), **L**1.2 > **A** (235), **P**114 > **G** (329)	L234A, L235A, P329G LALAPG	IGHG1 CH2 1.6–3 (231–239) APE**LL**GGPS > APE**AA**GGPS FG 105–117 (322–332) KVSNKA..L**P**API > KVSNKA..L**G**API	**ADCC reduction**. Reduces FcγR binding [121]	**CDC reduction**. Reduces C1q binding [121]	
**G1v14-67**	CH2 **A1.3,** **A1.2, ** **S27**	CH2 **L**1.3 > **A** (234), **L**1.2 > **A**(235), **D**27 > **S** (265)	L234A, L235A, D265S	IGHG1 CH2 1.6–3 (231–239) APE**LL**GGPS > APE**AA**GGPS 23–31 (261–269) CVVV**D**VSHE > CVVV**S**VSHE	**ADCC reduction**. Reduces FcγR binding [121].	**CDC reduction**. Reduces C1q binding [121].	Combines Homsap G1v14 and G1v67 (G1 CH2 S27).
**G1v23**	**CH2** **E1.2**	CH2 **L**1.2 > **E**(235)	L235E	IGHG1 CH2 1.6–3 (231–239) APEL**L**GGPS > APEL**E**GGPS	**ADCC reduction**. Reduces FcγR binding [122]	**CDC reduction**. Reduces C1q binding [122]	
**G1v38**	**CH2** **S108, ** **F113**	CH2 **N**108 > **S** (325), **L**113 > **F** (328)	N325S, L328F	IGHG1 CH2 FG 105–117 (322–332) KVS**N**KA..**L**PAPI > KVS**S**KA..**F**PAPI	**ADCC reduction**. Abrogates FcγRIII binding, increases FcγRII binding, retains FcγRI high affinity binding [123]	**CDC reduction**. Abrogates C1q binding.	
**G1v39**	**CH2** **F1.3, ** **E1.2, ** **S116**	CH2 **L**1.3 > F (234), **L**1.2 > **E** (235), **P**116 > **S** (331)	L234F, L235E, P331S FES, TM	IGHG1 CH2 1.6–3 (231–239) APE**LL**GGPS > APE**FE**GGPS FG 105–117 (322–332) KVSNKA..LPA**P**I > KVSNKA..LPA**S**I	**ADCC reduction** Reduces FcγR effector properties [124] (2)	**CDC reduction**. Reduces C1q binding [122]	3D 3c2s
**G1v40**	**CH2** **A1.3,** **A1.2,** **S116**	CH2 **L**1.3 > **A** (234), **L**1.2 > **A** (235), **P**116 > **S** (331)	L234A, L235A, P331S	IGHG1 CH2 1.6–3 (231–239) APE**LL**GGPS > APE**AA**GGPS FG 105–117 (322–332) KVSNKA..LPA**P**I > KVSNKA..LPA**S**I	**ADCC reduction**. Reduces FcγR binding.	**CDC reduction**. Reduces C1q binding.	
**G1v41**	**CH2** **F1.3,** **E1.2**	CH2 **L**1.3 > **F** (234), **L**1.2 > **E** (235)	L234F, L235E FE	IGHG1 CH2 1.6–3 (231–239) APE**LL**GGPS > APE**FE**GGPS	**ADCC reduction**. Reduces FcγR binding [124]	**CDC reduction**. Reduces C1q binding [122]	
**G1v43**	**CH2** **A1.3, ** **E1.2,** **A1 **	CH2 **L**1.3 > **A** (234), **L**1.2 > **E** (235), **G**1 > **A** (237)	L234A, L235E, G237A	IGHG1 CH2 1.6–3 (231–239) APE**LL**G**G**PS > APE**AE**G**A**PS	**ADCC reduction**. Reduces FcγR binding	**CDC reduction**. Reduces C1q binding	
**G1v48**	**CH2** **R113**	CH2 **L**113 > **R** (328)	L328R	IGHG1 CH2 FG 105–117 (322–332) KVSNKA..**L**PAPI > KVSNKA..**R**PAPI	**ADCC reduction**. Reduces FcγR binding	**CDC reduction**. Reduces C1q binding	
**G1v49**	**CH2** **G114**	CH2 **P**114 > **G** (329)	P329G	IGHG1 CH2 FG 105–117 (322–332) KVSNKA..L**P**API > KVSNKA..L**G**API	**ADCC reduction**. Reduces FcγR binding [121]	**CDC reduction**. Reduces C1q binding [121]	
**G1v51**	**CH2** **K29**	CH2 **S**29 > **K** (267)	S267K	IGHG1 CH2 27–31 (265–269) DV**S**HE > DV**K**HE	**ADCC reduction**. Reduces FcγR binding	**CDC reduction**. Reduces C1q binding	
**G1v53**	**CH2** **F1.3,** **Q1.2,** **Q105**	CH2 **L**1.3 > **F** (234) **L**1.2 > **Q** (235) **K**105 > **Q** (322)	L234F, L235Q, K322Q, FQQ	IGHG1 CH2 1.6–3 (231–239) APE**LL**GGPS > APE**FQ**GGPS FG 105–117 (322–332) **K**VSNKA..LPAPI > **Q**VSNKA..LPAPI	**ADCC reduction**. Reduces FcγR binding	**CDC reduction**. Reduces C1q binding	
**G1v53, G1v21**	**CH2** **F1.3,** **Q1.2,** **Q105** **Y15.1**, **T16,** **E18**	CH2 **L**1.3 > **F** (234), **L**1.2 > **Q** (235), **K**105 > **Q** (322) **M**15.1 > **Y** (252), **S**16 > **T** (254), **T**18 > **E** (256)	L234F, L235Q, K322Q, M252Y, S254T, T256E FQQ–YTE	IGHG1 CH2 1.6–3 (231–239) APE**LL**GGPS > APE**FQ**GGPS 15–18 (251–256) L**M**I.**S**R**T** > L**Y**I**T**R**E** FG 105–117 (322–332) **K**VSNKA..LPAPI > **Q**VSNKA..LPAPI	**ADCC reduction**. Reduces FcγR binding [125] (G1v53)	**CDC reduction**. Reduces C1q binding [125] (G1v53)	Extends half-life [125] (G1v21).
**G1v59**	**CH2** **S1.3** **T1.2** **R1.1**	CH2 **L**1.3 > **S**(234) **L**1.2 > **T** (235) **G**1.1 > **R** (236)	L234S L235T G236R	IGHG1 CH2 1.6–3 (231–239) APE**LLG**GPS > APE**STR**GPS	ADCC undetectable. Abrogates FcγR binding [126]	CDC undetectable. Abrogates C1q binding [126]	
**G1v60**	**CH2** **S115,** **S116**	CH2 **A**115 > **S**(330) **P**116 > **S** (331)	A330S P331S	FG 105–117 (322–332) KVSNKA..LP**AP**I > QVSNKA..LP**SS**I	**ADCC reduction**. Reduces FcγR binding.	**CDC reduction**. Reduces C1q binding.	
**G1v63**	**CH2** **S2**	CH2 **P**2 > **S**	P238S	IGHG1 CH2 1.6–3 (231–239) APELLGG**P**S > APELLGG**S**S	**ADCC reduction**. Reduces FcγR binding.	**CDC reduction**. Reduces C1q binding.	
**G1v65**	**CH2** **delE1.4,** **delL1.3,** **delL1.2**	CH2 **E**1.4 > **del**, **L**1.3 > **del**, **L**1.2 > **del**	E233del, L234del, L235del	IGHG1 CH2 1.6–3 (231–239) AP**ELL**GGPS > AP**- - -**GGPS	**ADCC reduction**. Reduces FcγR binding.	**CDC reduction**. Reduces C1q binding.	
**G1v70**	**h** **S5,** **S11,** **S14,** **CH2** **S2**	h **C**5 > **S**(220), **C**11 > **S** (226) **C**14 > **S**(226) CH2 P2 > **S**	C220S C226S C229S P238S	IGHG1 h 1–15 (216–230) EPKS**C**DKTHT**C**PP**C**P > EPKS**S**DKTHT**S**PP**S**P IGHG1 CH2 1.6–3 (231–239) APELLGG**P**S > APELLGG**S**S	**ADCC reduction**. Reduces FcγR binding.	**CDC reduction**. Reduces C1q binding.	Combines G1v63 with G1v37 (no H-L), G1v61 (no H-H h11) and G1v62 (no H-H h14).
**G2v2**	**CH2** **Q30, ** **L92, ** **S115**, **S116**	CH2 **H**30 > **Q**(268), **V**92 > **L**(309), **A**115 > **S**(330), **P**116 > **S**(331)	H268Q, V309L, A330S, P331S IgG2m4	IGHG2 CH2 27–38 (265–274) DVS**H**EDPEVQ > DVS**Q**EDPEVQ 89–96 (306–313) LTV**V**HQDW > LTV**L**HQDW FG 105–117 (322–332) KVSNKG..LP**AP**I > KVSNKA..LP****SS****I****	**ADCC reduction**. Reduces FcγR binding [127]	**CDC reduction**. Reduces C1q binding [127]	
**G2v3**	**CH2** **A1.2,** **A1, ** **S2, ** **A30, ** **L92, ** **S115,** **S116**	CH2 **V**1.2 > A (235), **G**1 > A (237), **P**2 > **S**(238), **H**30 > **A**(268), **V**92 > **L**(309), **A**115 > **S**(330), **P**116 > **S**(331)	V235A, G237A, P238S, H268A, V309L, A330S, P331S G2sigma	IGHG2 CH2 1.6–3 (231–239) AP.P**V**A**GP**S > AP.P**A**A**AS**S 27–38 (265–274) DVS**H**EDPEVQ > DVS**A**EDPEVQ 89–96 (306–313) LTV**V**HQDW > LTV**L**HQDW FG 105–117 (322–332) KVSNKG..LP**AP**I > KVSNKA..LP****SS****I****	**ADCC reduction**. Reduces FcγR binding [124]. Undetectable ADCC andV1 ADCP [124]	**CDC reduction**. Reduces C1q binding [124]. Undetectable CDC [124]	
**G2G4v1** **(1)**	**CH2** **E1.4 > del** **P1.3**, **V1.2,** **A1.1**	CH2 **E**1.4 > **del**(233), **F**1.3 > **P**(234), **L**1.2 > **V**(235), **G**1.1 > **A**(236)	E233del, F234P, L235V, G236A	IGHG4 CH2 1.6–3 (231–239) AP**EFLG**GPS > AP.**PVA**GPS	**ADCC reduction**. Reduces FcγR binding [128]	**CDC reduction**. Reduces C1q binding [128]	
**G4v3**	**CH2** **E1.2**	CH2 **L**1.2 > **E**(235)	L235E LE	IGHG4 CH2 1.6–3 (231–239) APEF**L**GGPS > APEF**E**GGPS	**ADCC reduction**. Reduces FcγR binding [122]	**CDC reduction**. Reduces C1q binding [122]	
**G4v3** **G4v5**	**h** **P10,** **CH2** **E1.2**	h **S**10 > **P**(228) CH2 **L**1.2 > **E**(235)	S228P, L235E SPLE	IGHG4 h 1–12 (216–230) ESKYGPPCP**S**CP > ESKYGPPCP**P**CP CH2 1.6–3 (231–239) APEF**L**GGPS > APEF**E**GGPS	**ADCC reduction**. Reduces FcγR binding [122] (G4v3)	**CDC reduction**. Reduces C1q binding [122] (G4v3)	Prevents IgG4 half-IG exchange [129] (G4v5)
**G4v3-49**	**CH2** **E1.2** **G114**	CH2 **L**1.2 > **E**(235) **P**114 > **G** (329)	L235E P329G LEPG	IGHG4 CH2 1.6–3 (231–239) APEF**L**GGPS > APEF**E**GGPS FG 105–117 (322–332) KVSNKA..L**P**API > KVSNKA..L**G**API	**ADCC reduction**. Reduces FcγR binding [121]	**CDC reduction**. Reduces C1q binding [121]	
**G4v3-49** **G4v5**	**h** **P10,** **CH2** **E1.2** **G114**	h **S**10 > **P**(228) CH2 **L**1.2 > **E**(235) **P**114 > **G** (329)	S228P, L235E P329G SPLEPG	IGHG4 h 1–12 (216–230) ESKYGPPCP**S**CP ESKYGPPCP**P**CP CH2 1.6–3 (231–239) APEF**L**GGPS > APEF**E**GGPS FG 105–117 (322–332) KVSNKA..L**P**API > KVSNKA..L**G**API	**ADCC reduction**. Reduces FcγR binding [121] (G4v3-49)	**CDC reduction**. Reduces C1q binding [121] (G4v3-49)	Prevents IgG4 half-IG exchange [129] (G4v5).
**G4v4**	**CH2** **A1.3, ** **A1.2**	CH2 **F**1.3 > **A** (234), **L**1.2 > **A** (235)	F234A L235A FALA	IGHG4 CH2 1.6–3 (231–239) APE**FL**GGPS > APE**AA**GGPS	**ADCC reduction**. Reduces FcγR binding [120].	**CDC reduction**. Reduces C1q binding [120].	
**G4v4** **G4v5**	**h** **P10,** **CH2** **A1.3,** **A1.2**	h **S**10 > **P**(228) CH2 **F**1.3 > **A** (234) **L**1.2 > **A** (235)	S228P, F234A, L235A IgG4 ProAlaAla	IGHG4 h 1–12 (216–230) ESKYGPPCP**S**CP > ESKYGPPCP**P**CP CH2 1.6–3 (231–239) APE**FL**GGPS > APE**AA**GGPS	**ADCC reduction**. Reduces FcγR binding [124] (G4v4)	**CDC reduction**. Reduces C1q binding [120] (G4v4)	Prevents IgG4 half-IG exchange [129] (G4v5).
**G4v7**	**CH2** **delE1.4,** **P1.3, ** **V1.2, ** **A1.1**	CH2 **E**1.4 > del (233) **F**1.3 > **P** (234), **L**1.2 > **V** (235), **G**1.1 > **A** (236),	E233del, F234P, L235V, G236A	IGHG4 CH2 1.6–3 (231–239) AP**EFLG**GPS> AP**-PVA**GPS (G2-like)	**ADCC reduction**. Reduces FcγR binding	**CDC reduction**. Reduces C1q binding	
**G4v49**	**CH2** **G114**	CH2 **P**114 > **G** (329)	P329G	IGHG4 CH2 FG 105–117 (322–332) KVSNKA..L**P**API > KVSNKA..L**G**API	**ADCC reduction**. Reduces FcγR binding [121]	**CDC reduction**. Reduces C1q binding [121]	
***Canis lupus familiaris*** **G2v1**	**CH2** **A1.3, ** **A1.2, ** **A1**	CH2 **M**1.3 > **A** (234), **L**1.2 > **A** (235), **G**1 > **A** (237).	M234A, L235A, G237A	IGHG2 CH2 1.6–3 (231–239) APE**ML**G**G**PS > APE**AA**G**A**PS	**ADCC reduction**. Reduces FcγR binding	**CDC reduction**. Reduces C1q binding	
***Canis lupus familiaris*** **G2v2**	**CH2** **A1.3, ** **A1.2,** **G114**	CH2 **M**1.3 > **A** (234), **L**1.2 > **A**(235) **P**114 > **G**(329)	M234A, L235A, P329G	IGHG2 CH2 1.6–3 (231–239) APE**ML**GGPS > APE**AA**GGPS IGHG1 CH2 FG 105–117 (322–332) KVNNKA..L**P**SPI > KVNNKA..L**G**SPI	**ADCC reduction**. Reduces FcγR binding	**CDC reduction**. Reduces C1q binding	

(1) The monoclonal antibody is eculizumab. The heavy chain is the chimeric IGHG2*01 CH1-hinge—IGHG4*01 CH2-CH3. The CH2 and CH3 are from IGHG4*01, except for the CH2 positions 1.6-1.1 (AP.PVA) with del 1.4 and amino acids P1.3, V1.2 and A1.1 being from IGHG2*01. The changes are shown in comparison to the IGHG4*01 amino acids at the same positions as E1.4, F1.3, L1.2 and G1.1.

**Table 11:** IMGT nomenclature, Eu positions and IMGT motif of engineered Fc variants involved in the B cell inhibition by the coengagement of antigen and FcγR on the same cell (Effector #7].

IMGT Engineered Fc Variant Name	IMGT Engineered Variant Definition	IMGT Amino Acid Changes on IGHG CH Domain (Eu Numbering between Parentheses)	Amino Acid Changes with the Eu Positions	Motif Identifiable in Gene and Domain with Positions According to the IMGT Unique Numbering and with Eu Positions between Parentheses	Property and Function
**G1v25**	**CH2** **E29,** **F113**	CH2 **S**29 > **E** (267), **L**113 > **F** (328)	S267E, L328F	IGHG1 CH2 27–31 (265–269) DV**S**HE > DV**E**HE FG 105–117 (322–332) KVSNKA..****L****PAP****I** >** KVSNKA..****F****PAPI	Increases FcγRIIB binding (400-fold) [130] Inhibits by downstream ITIM signaling in B cells [131]

**Table 12:** IMGT nomenclature, Eu positions and IMGT motif of engineered Fc variants involved in the knock out CH2 84.4 glycosylation (Effector #8).

IMGT Engineered Variant Name	IMGT Engineered Variant Definition	IMGT Amino Acid Changes on IGHG CH Domain (Eu Numbering between Parentheses)	Amino Acid Changes with the Eu Positions	Motif Identifiable in Gene and Domain with Positions According to the IMGT Unique Numbering and with Eu Positions between Parentheses	Property and Function
**G1v29**	**CH2** **A84.4**	CH2 **N**84.4 > **A** (297)	N297A	IGHG1 CH2 83–86 REEQY**N**STYRVV > REEQY**A**STYRVV	**ADCC reduction**. Reduces FcγR binding [132]
**G1v30**	**CH2** **G84.4**	CH2 **N**84.4 > **G**(297)	N297G	IGHG1 CH2 83–86 REEQY**N**STYRVV > REEQY**G**STYRVV	**ADCC reduction**. Reduces FcγR binding [132]
**G1v36**	**CH2** **Q84.4**	CH2 **N**84.4 > **Q** (297)	N297Q	IGHG1 CH2 83–86 REEQY**N**STYRVV > REEQY**Q**STYRVV	**ADCC reduction**. Reduces FcγR binding
**G4v36**	**CH2** **Q84.4**	CH2 **N**84.4 > **Q** (297)	N297Q	IGHG4 CH2 83–86 REEQF**N**STYRVV > REEQF**Q**STYRVV	**ADCC reduction**. Reduces FcγR binding
***Canis lupus familiaris*** **G2v29**	**CH2** **A84.4**	CH2 **N**84.4 > **A** (297)	N297A	IGHG1 CH2 83–86 RE**EQF****N****GTYR**VV > RE**EQF****A****GTYR**VV	**ADCC reduction**. Reduces FcγR binding

**Table 13:** IMGT nomenclature, Eu positions and IMGT motif of engineered Fc variants involved in half-life increase (Half-life #9).

IMGT Engineered Variant Name	IMGT Engineered Variant Definition	IMGT Amino Acid Changes on IGHG CH Domain (Eu Numbering between Parentheses)	Amino Acid Changes with the Eu Positions	Motif Identifiable in Gene and Domain with Positions According to the IMGT Unique Numbering and with Eu Positions between Parentheses	Property and Function
**G1v21**	**CH2** **Y15.1,** **T16,** **E18**	CH2 **M**15.1 > **Y** (252), **S**16 > **T** (254), **T**18 > **E** (256)	M252Y, S254T, T256E YTE	IGHG1 CH2 13–18 (249–256) DTL**M**I**S**R**T** > DTL**Y**I**T**R**E**	**Half-life increase** Enhances FCGRT binding at pH 6.0 [133,134] (1)
**G1v22**	**CH2** **Y15.1, ** **T16, ** **E18**, **CH3** **K113, ** **F114, ** **H116**	CH2 **M**15.1 > **Y** (252) **S**16 > **T** (254) **T**18 > **E** (256) CH3 **H**113 > **K** (433) **N**114 > **F** (434) **Y**116 > **H** (436)	M252Y S254T T256E H433K N434F Y436H	IGHG1 CH2 13–18 (249–256) DTL**M**I**S**R**T** > DTL**Y**I**T**R**E** CH3 FG 105–117 (426–437) SVMHEA.L**HN**H**Y**T > SVMHEA.L**KF**H**H**T	**Half-life increase** Enhances FCGRT binding at pH 6.0 [134]
**G1v24**	**CH3** **L107, ** **S114**	CH3 **M**107 > **L** (428), **N**114 > **S** (434)	M428L, N434S	GHG1 CH3- FG 105–117 (426–437) SV**M**HEA.LH**N**HYT > SV**L**HEA.LH**S**HYT	**Half-life increase** Enhances FCGRT binding at pH 6.0 (11-fold increase in affinity) [135] (2)
**G1v42**	**CH2** **Q14**, **CH3** **L107**	CH2 T14 > Q (250) CH3 M107 > L (428)	T250Q M428L	IGHG1 CH2 13–18 (249–256) D**T**LMISRT > D**Q**LMISRT CH3- FG 105–117 (426–437) SV**M**HEA.LHNHYT > SV**L**HEA.LHNHYT	**Half-life increase** Enhances FCGRT binding at pH 6.0 [134]
**G1v46**	**CH3** **K113,** **F114**	CH3 **H**113 > **K** (433), **N**114 > **F**(434)	H433K, N434F	IGHG1 CH3- FG 105–117 (426–437) SVMHEA.L**HN**HYT > SVMHEA.L**KF**HYT	**Half-life increase** Enhances FCGRT binding at pH 6.0.
**G2v4**	**CH2** **Q14**	CH2 **T**14 > **Q** (250)	T250Q	IGHG2 CH2 13–18 (249–256) D**T**LMISRT > D**Q**LMISRT	**Half-life increase** Enhances FCGRT binding at pH 6.0 [136]
**G2v5**	**CH3** **L107**	CH3 **M**107 > **L** (428)	M428L	IGHG2 CH3 FG 105–117 (426–437) SV****M****HEA.LHNHYT > SV****L****HEA.LHNHYT	**Half-life increase** Enhances FCGRT binding at pH 6.0 [136]
**G2v6**	**CH2** **Q14**, **CH3** **L107**	CH2 **T**14 > **Q** (250) CH3 **M**107 > **L**(428)	T250Q M428L	IGHG2 CH2 13–18 (249–256) D**T**LMISRT > D**Q**LMISRT CH3 FG 105–117 (426–437) SV****M****HEA.LHNHYT > SV****L****HEA.LHNHYT	**Half-life increase** Enhances FCGRT binding at pH 6.0 [136]
**G2v8-1**	**CH2** **A93**	CH2 **H**93 > **A** (310)	H310A	IGHG2 CH2 89–96 (306–313) LTVV****H****QDW > LTVV**A**QDW	**Abrogates FCGRT binding at pH 6.0** (G2v8 any amino acid replacement of H93 except cystein) [137]. Number 1 of G2v8-1 is for A
**G3v1**	**CH3** **H115**	CH3 **R**115 > **H**(435)	R435H	IGHG3 CH3 FG 105–117 (426–437) SVMHEA.LHN****R****FT > SVMHEA.LHN****H****FT	**Half-life increase** Extends half-life [138]
**G4v21**	**CH2** **Y15.1,** **T16, ** **E18**	CH2 **M**15.1 > **Y** (252), **S**16 > T (254), **T**18 > **E** (256)	M252Y, S254T, T256E YTE	IGHG4 CH2 13–18 (249–256) DTL**M**I**S**R**T** > DTL**Y**I**T**R**E**	**Half-life increase** Enhances FCGRT binding at pH 6.0 [134]
**G4v22**	**CH2** **T16,** **P91**, **CH3** **A114**	CH2 **S**16 > **T**(254), **V**91 > **P** (308) CH3 **N**114 > **A** (434)	S254T, V308P N434A	IGHG4 CH2 13–18 (249–256) DTL**M**I**S**R**T** > DTL**Y**I**T**R**E** CH3 FG 105–117 (426–437) SVMHEA.LH****N****HYT > SVMHEA.LH****A****HYT	**Half-life increase** Enhances FCGRT binding at pH 6.0 [139]
**G4v24**	**CH3** **L107** **S114**	CH3 **M**107 > **L** (428) **N**114 > **S**(434)	M428L, N434A	CH3 FG 105–117 (426–437) SV**M**HEA.LH**N**HYT > SV**L**HEA.LH**S**HYT	**Half-life increase** Enhances FCGRT binding at pH 6.0

(1) Ten-fold increase at pH 6.0 [134] and four-fold increases half-life in a cynomolgus pK study [140]. The T18>E amino acid change provides two novel salt bridges between the Fc and ΒM2 of FCGRT IMGT/3Dstructure-DB: 4n0f, 4n0u [137]. A change of IGHG1 CH2 His H93 (310) into any other amino acid (excluding Cys) leads to an undetectable binding to FCGRT (FcRn) at pH 6.0 [137]. (2) An increased reduction in tumor burden in human FCGRT (FcRn) transgenic tumor-bearing mice treated with an anti-EGFR or an anti-VEGF antibody [135]. From the 3D structure, it is postulated that N114>S (434) allows for additional hydrogen bonds with FCGRT (FcRn) [137] IMGT/3Dstructure-DB: 4n0f, 4n0u.

**Table 14:** IMGT nomenclature, Eu positions and IMGT motif of engineered Fc variants involved in the abrogation of binding to Protein A (Protein A #10).

IMGT Engineered Variant Name	IMGT Engineered Variant Definition	IMGT Amino acid changes on IGHG CH domain (Eu numbering between parentheses)	Amino acid changes with the Eu positions	Motif identifiable in gene and domain with positions according to the IMGT unique numbering and with Eu positions between parentheses	Property and function
**G4v8**	**CH3** **R115, ** **F116, ** **P125**	CH3 **H**115 > **R** (435), **Y**116 > **F** (436), **L**125 > **P** (445)	H435R, Y436F, L445P	IGHG4 CH3- FG 105–117 (426–437) SVMHEA.LHN**HY**T > SVMHEA.LHN**RF**T 118–125 (438–445) QKSLSLS**L** > QKSLSLS**P**	Abrogates binding to Protein A

**Table 15:** IMGT nomenclature, Eu positions and IMGT motif of engineered Fc variants involved in the formation of additional bridge stabilizing CH2 in the absence of N84.4 (297) glycosylation (Structure #11).

IMGT Engineered Variant Name	IMGT Engineered Variant Definition	IMGT Amino Acid Changes on IGHG CH Domain (Eu Numbering between Parentheses)	Amino Acid Changes with the Eu Positions	Motif Identifiable in Gene and Domain with Positions According to the IMGT Unique Numbering and with Eu Positions between Parentheses	Property and Function
**G1v54**	**CH2** **C83,** **C85**	CH2 **R**83 > **C** (292), **V**85 > **C** (302)	R292C, V302C	IGHG1 CH2 83–86 REEQYNSTYRVV > CEEQY**A**STYRCV (v29) CEEQY**G**STYRCV (v30) CEEQY**Q**STYRCV (v36)	Stabilizes CH2 in the absence of N84.4 (297) glycosylation
**G1v54-29**	**CH2** **C83, ** **A84.4,** C85	CH2 **R**83 > **C** (292), **N**84.4 > **A**(297) **V**85 > **C** (302)	R292C, N297A V302C	IGHG1 CH2 83–86 REEQY**N**STYRVV > CEEQY**A**STYRCV	Stabilizes CH2 in the absence of N84.4 (297) glycosylation
**G1v54-30**	**CH2** **C83, ** **G84.4,** **C85**	CH2 **R**83 > **C** (292), **N**84.4 > **G** (297) **V**85 > **C** (302)	R292C, N297G V302C	IGHG1 CH2 83–86 REEQY**N**STYRVV > CEEQY**G**STYRCV	Stabilizes CH2 in the absence of N84.4 (297) glycosylation
**G1v54-36**	**CH2** **C83, ** **Q84.4,** **C85**	CH2 **R**83 > **C** (292), **N**84.4 > **Q** (297) **V**85 > **C** (302)	R292C, N297Q V302C	IGHG1 CH2 83–86 REEQY**N**STYRVV > CEEQYQSTYRCV	Stabilizes CH2 in the absence of N84.4 (297) glycosylation

**Table 16:** IMGT nomenclature, Eu positions and IMGT motif of engineered Fc variants involved in the prevention of IgG4 half-IG exchange (Structure #12).

IMGT Engineered Variant Name	IMGT Engineered Variant Definition	IMGT Amino Acid Changes on IGHG CH Domain (Eu Numbering between Parentheses)	Amino Acid Changes with the Eu Positions	Motif Identifiable in Gene and Domain with Positions According to the IMGT Unique Numbering and with Eu Positions between Parentheses	Property and Function
**G4v5**	**h** **P10**	h **S**10 > **P**(228)	S228P	IGHG4 h 1–12 (216–230) ESKYGPPCP**S**CP > ESKYGPPCP**P**CP (G1-like)	Prevents in vivo and in vitro IgG4 half-IG exchange [129]
**G4v6**	**CH3** **K88**	**CH3** **R**88 > **K**	R409K	IGHG1 CH3 85.4–89 (404–410) GSFFLYS**R**L > GSFFLYS**K**L	Reduces IgG4 half-IG exchange [141]

**Table 17:** IMGT nomenclature, Eu positions and IMGT motif of engineered Fc variants involved in hexamerisation (Structure #13).

IMGT Engineered Variant Name	IMGT Engineered Variant Definition	IMGT Amino Acid Changes on IGHG CH Domain (Eu Numbering between Parentheses)	Amino Acid Changes with the Eu Positions	Motif Identifiable in Gene and Domain with Positions According to the IMGT Unique Numbering and with Eu Positions between Parentheses	Property and Function
**G1v34**	**CH3** **G109**	CH3 **E**109 > **G** (430)	E430G	IGHG1 CH3- FG 105–117 (426–437) SVMH**E**A.LHNHYT > SVMH**G**A.LHNHYT	Favors IgG1 hexamerisation by increased intermolecular Fc-Fc interactions after antigen binding on the cell surface

**Table 18:** IMGT nomenclature, Eu positions and IMGT motif of engineered Fc variants involved in knobs-into-holes and the enhancement of heteropairing H-H of bispecific antibodies (Structure #14).

IMGT Engineered Variant Name	IMGT Engineered Variant Definition	IMGT Amino Acid Changes on IGHG CH Domain (Eu Numbering between Parentheses)	Amino Acid Changes with the Eu Positions	Motif Identifiable in Gene and Domain with Positions According to the IMGT Unique Numbering and with Eu Positions between Parentheses	Property and Function
**G1v26**	**CH3** **Y22**	CH3 **T**22 > **Y** (366)	T366Y	IGHG1 CH3 20–26 (364–370) SL**T**CLVK > SL**Y**CLVK	**Knob** of knobs-into-holes G1v26 knob/G1v31 hole interactions between the CH3 of the two different gamma1 chains [142]
**G1v31**	**CH3** **T86**	CH3 **Y**86 > **T** (407)	Y407T	IGHG1 CH3 85.4–89 (404–410) GSFFL**Y**SKL > GSFFL**T**SKL	**Hole** of knobs-into-holes G1v26 knob/G1v31 hole interactions between the CH3 of the two different gamma1 chains [142] (G1v26 knob/G1v31 hole)
**G1v32**	**CH3** **W22**	CH3 **T**22 > **W** (366)	T366W	IGHG1 CH3 20–26 (364–370) SL**T**CLVK > SL**W**CLVK	**Knob** of knobs-into-holes G1v32 knob/G1v33 hole interactions between the CH3 of the two different gamma1 chains
**G1v33**	**CH3** **S22, ** **A24, ** **V86**	CH3 **T**22 > **S** (366), **L**24 > **A** (368), **Y**86 > **V**(407)	T366S, L368A, Y407V	IGHG1 CH3 20–26 (364–370) SL**T**C**L**VK > SL**S**C**A**VK 85.4–89 (404–410) GSFFL**Y**SKL> GSFFL**V**SKL	**Hole** of knobs-into-holes G1v32 knob/G1v33 hole interactions between the CH3 of the two different gamma1 chains
**G1v68**	**CH3** **V6, ** **L22, ** **L79, ** **W81**	CH3 **T**6 > **V** (350) **T**22 > **L** (366) **K**79 > **L** (392) **T**81 > **W** (394)	T350V T366L K392L T394W	IGHG1 CH3 3–9 (347–353) QVY**T**LPP > QVY**V**LPP 20–26 (364–370) SL**T**CLVK > SL**L**CLVK 77–83 (390–396) NY**K**T**T**PP > NY**L**T**W**PP	Enhances, with G1v69, the heteropairing H-H of bispecific antibodies
**G1v69**	**CH3** **V6,** **Y7,** **A85.1,** **V86**	CH3 **T**6 > **V** (350) **L**7 > **Y** (351) **F**85.1 > **A** (405) **Y**86 > **V** (407)	T350V L351Y F405A Y407V	IGHG1 CH3 3–9 (347–353) QVY**TL**PP > QVY**VY**PP IGHG1 CH3 85.4–89 (404–410) GSF**F**L**Y**SKL > GSF**A**L**V**SKL	Enhances, with G1v68, the heteropairing H-H of bispecific antibodies

**Table 19:** IMGT nomenclature, Eu positions and IMGT motif of engineered Fc variants involved in the suppression of inter H-L and/or inter H-H disulfide bridges (Structure #15).

IMGT Variant Name	IMGT Variant Description	IMGT Amino Acid Changes on IGHG CH Domain with Eu Numbering between Parentheses	Eu Numbering Variant	Motif Identifiable in Gene and Domain with Positions According to the IMGT Unique Numbering	Property and Function
**G1v37**	**h** **S5**	h **C**5 > **S** (220)	C220S	IGHG1 h 1–15 (216–230) EPKS**C**DKTHTCPPCP > EPKS**S**DKTHTCPPCP	No disulfide bridge inter H-L
**G1v61**	**h** **S11**	h **C**11 > **S** (226)	C226S	IGHG1 h 1–15 (216–230) EPKSCDKTHT**C**PPCP > EPKSCDKTHT**S**PPCP	No disulfide bridge inter H-H h 11
**G1v62**	**h** **S14**	h **C**14 > **S** (229)	C229S	IGHG1 h 1–15 (216–230) EPKSCDKTHTCPP**C**P > EPKSCDKTHTCPP**S**P	No disulfide bridge inter H-H h 14

**Table 20:** IMGT nomenclature, Eu positions and IMGT motif of engineered Fc variants involved in site-specific drug attachment (Structure #16).

IMGT Variant Name	IMGT Variant Description	IMGT Amino Acid Changes on IGHG CH Domain with Eu Numbering between Parentheses	Eu Numbering Variant	Motif Identifiable in Gene and Domain with Positions According to the IMGT Unique Numbering	Property and Function
**G1v27**	**CH2** **C3**	CH2 **S**3 > **C**(329)	S239C	IGHG1 CH2 1.6–4 (231–240) APELLGGP**S**V > APELLGGP**C**V	Site-specific drug attachment engineered cysteine
**G1v28**	**CH2** **C(3^4)**	CH2 (3^4)**C**(239^240)	C(239^240)	IGHG1 CH2 1.6–4 (231–240) APELLGGPSV > APELLGGPS**C**V	Site-specific drug attachment engineered cysteine
**G1v44**	**CH3** **C122**	CH3 **S**122 > C (442)	S442C	IGHG1 CH3 118–125 (438–445) QKSL**S**LSP > QKSL**C**LSP	Site-specific drug attachment engineered cysteine
**G1v55**	**CH3** **C123**	CH3 **L**123 > **C** (443)	L443C	IGHG1 CH3 118–125 (438–445) QKSLS**L**SP > QKSLS**C**SP	Site-specific drug attachment engineered cysteine
**G1v56**	**CH2** **F85.2** **CH3** **F85.2**	CH2 **Y**85.2 > **F** (pAMF) CH3 **F**85.2 > **F** (pAMF)	Y300F F404F	IGHG1 CH2 84.1–85.1 (294–301) EQYNST**Y**R > EQYNST**F**R CH3 84.1–85.1 (398–405) LDSDGSFF LDSDGSFF	Modified phenylalanine for conjugation (produced in *Escherichia coli*, non glycosylated)
**G1v64**	**CH2** **C36**	CH2 **E**36 > **C**	E272C	IGHG1 CH2 34–41 (270–277) DP**E**VKFNW > DP**C**VKFNW	Conjugation site-specific engineered cysteine

**Table 21:** IMGT nomenclature, Eu positions and IMGT motif of engineered Fc variants involved in the enhancement of hetero pairing H-L of bispecific antibodies (Structure #17).

IMGT Variant Name	IMGT Variant Description	IMGT Amino Acid Changes on IGHG CH Domain with Eu Numbering between Parentheses	Eu Numbering Variant	Motif Identifiable in Gene and Domain with Positions According to the IMGT Unique Numbering	Property and Function
**G1v57**	**CH1** **E26, ** **E119**	CH1 **K**26 > **E** (147), **K**119 > **E**(213)	K147E, K213E	IGHG1 CH1 23–26 (144–147) CLV**K** > CLV**E** 118–121 (212–215) D**K**KV > D**E**KV	Enhances, with KCv57, the hetero pairing H-L of bispecific antibodies
**KCv57**	**IGKC** **R12, ** **K13**	IGKC **E**12 > **R**, **Q**13 > **K**	E123R, Q124K	IGKC 10–15 (121–126) SD**EQ**LK > SD**RK**LK	Enhances, with G1v57, the hetero pairing H-L of bispecific antibodies
**G1v58**	**CH1** **C5,** **h** **V5**	CH1 **F**5 > **C** (126), h **C**5 > **V** (220)	F126C, C220V	IGHG1 CH1 1.4–15 (118–136) ASTKGPSV****F****PLAPSSKSTS > ASTKGPSV****C****PLAPSSKSTS IGHG1 h 1–15 (216–230) EPKS**C**DKTHTCPPCP > EPKS**V**DKTHTCPPCP	Alternative interchain cysteine mutations to enhance, with LC2v58, heteropairing of bispecific antibodies
**LC2v58**	**LC2** **C10, ** **V126**	IGLC **S**10 > **C** (121), **C**126 > **V** (214)	S121C, C214V	IGLC2 1.5–15 (107A–126) GQPKAAPSVTLFPP**S**SEELQ > GQPKAAPSVTLFPP**C**SEELQ IGLC2 118–127 (206–215) EKTVAPTE**C**S > EKTVAPTE**V**S	Alternative interchain cysteine mutations to enhance, with G1v58, heteropairing of bispecific antibodies

**Table 22:** IMGT nomenclature, Eu positions and IMGT motif of engineered Fc variants involved in the control of half-IG exchange of bispecific IgG4 (Structure #18).

IMGT Variant Name	IMGT Variant Description	IMGT Amino Acid Changes on IGHG CH Domain with Eu Numbering between Parentheses	Eu Numbering Variant	Motif Identifiable in Gene and Domain with Positions According to the IMGT Unique Numbering	Property and Function
**G4v10**	**CH3** **L85.1, ** **K88**	CH3 **F**85.1 > **L**(405), **R**88 > **K** (409)	F405L, R409K	IGHG1 CH3 85.4–92 (402–413) GSF**F**LYS**R**LTVD > GSF**L**LYS**K**LTVD	Control of half-IG exchange of bispecific IgG4

**Table 23:** IMGT nomenclature, Eu positions and IMGT motif of engineered Fc variants involved in reducing acid-induced aggregation (Structure #19).

IMGT Engineered Fc Variant Name	IMGT Engineered Variant definition	IMGT Amino Acid Changes on IGHG CH Domain (Eu Numbering between Parentheses)	Amino Acid Changes with the Eu Positions	Motif Identifiable in Gene and Domain with Positions According to the IMGT Unique Numbering and with Eu Positions between Parentheses	1. Property and Function	2. Property and Function	3. Property and Function
**G2v7**	**CH2** **Y85.2,** **L92,** **A339**	CH2 **F**85.2 > **Y**(300) **V**92 > **L**(309) **T**339 > **A**(339)	F300Y V309L T339A	IGHG2 CH2 85.4–92 (300–309) ST**F**RVVSVLTV**V** > ST**Y**RVVSVLTV**L** 118–125 (333–340) EKTISK**T**K > EKTISK**A**K	Reduces acid-induced aggregation [143]	**Low ADCC** Low FcγR binding [143]	**Low CDC** Low C1q binding [143]

## Data Availability

Data is contained within the article or Supplementary material.

## Supplementary Materials

The following supporting information can be downloaded at: https://www.mdpi.com/article/10.3390/antib11040065/s1, Table S1: Correspondence between the IMGT unique numbering for C-DOMAIN, the IMGT exon numbering, the EU and Kabat numberings: Human IGHG [97,98] https://www.imgt.org/IMGTScientificChart/Numbering/Hu_IGHGnber.html; Table S2: IMGT nomenclature (alphanumeric order) of engineered variants involved in effector properties (ADCC, ADCP, CDC), half-life and structure of therapeutical monoclonal antibodies.
