# Supplementary material for: IMGT® Nomenclature of Engineered IGHG Variants Involved in Antibody Effector Properties and Formats
Source: Antibodies (Basel). 2022 Oct 18;11(4):65. doi: 10.3390/antib11040065 (PMC9624366; doi:10.3390/antib11040065)
Supplement: Supplementary file 1 [file antibodies-11-00065-s001.zip › antibodies-1895207-supplementary.pdf]

Supplementary material

Table S1. Correspondence between the IMGT unique numbering for C-DOMAIN, the IMGT exon numbering, the EU and Kabat numberings: Human IGHG [1,2] https://www.imgt.org/IMGTScientificChart/Numbering/Hu_IGHGnber.html.

Table S2. IMGT nomenclature (alphanumeric order) of engineered variants involved in effector properties (ADCC, ADCP, CDC), half-life and structure of therapeutical monoclonal antibodies.

Type	Species	IMGT engineered Fc variant name	IMGT engineered variant definition	IMGT amino acid changes on IGHG CH domain (Eu numbering between parentheses)	Amino acid changes with the Eu positions	Motif identifiable in gene and domain with positions according to the IMGT unique numbering and with Eu positions between parentheses	1. Property and function	2. Property and function	3. Property and function /3D
**1**	**Homsap**	**G1v1**	**CH2**<br>**P1.4**	CH2<br>**E**1.4>**P** (233)	E233P	IGHG1 CH2<br>1.6-3 (231-239)<br>APELLGGPS ><br>APPLLGGPS	**ADCC reduction**.<br>Prevents FcγRI binding [3]		
**1**	**Homsap**	**G1v2**	**CH2**<br>**V1.3**	CH2<br>**L**1.3>**V** (234)	L234V	IGHG1 CH2<br>1.6-3 (231-239)<br>APELLGGPS ><br>APEVLGGPS	**ADCC reduction**. Decreases FcγRI binding [3]		
**1**	**Homsap**	**G1v3**	**CH2**<br>**A1.2**	CH2<br>**L**1.2>**A** (235)	L235A	IGHG1 CH2<br>1.6-3 (231-239)<br>APELLGGPS ><br>APELAGGPS	**ADCC reduction**.<br>Prevents FcγRI binding [3]		
**6**	**Homsap**	**G1v4**	**CH2**<br>**A114**	**CH2**<br>**P**114>**A** (329)	P329A)	IGHG1 CH2<br>FG 105-117 (322-332)<br>KVSNKA..LPAPI ><br>KVSNKA..LAAPI	**ADCC reduction**.<br>Reduces FcγR binding [4]	**CDC reduction**.<br>Reduces C1q binding [4]	
**1, 4**	**Homsap**	**G1v5**	**CH2**<br>**W109**	**CH2**<br>**K**109>**W** (326)	K326W	IGHG1 CH2 FG 105-117 (322-332)<br>KVSNKA..LPAPI ><br>KVSNWA..LPAPI	**ADCC reduction** [5]	**CDC enhancement**.<br>Increases C1q binding [5]	
**2**	**Homsap**	**G1v6**	**CH2**<br>**A85.4, A118, A119**	CH2<br>**S**85.4>**A** (298),<br>**E**118>**A** (333), **K**119>**A** (334)	S298A,<br>E333A,<br>K334A	IGHG1 CH2<br>84.1-85.1 (294-301)<br>EQYNSTYR ><br>EQYNATYR<br>FG 105-117,118,119<br>(322-334)<br>KVSNKA..LPAPIEK ><br>KVSNKA..LPAPIAA	**ADCC enhancement**.<br>Increases FcγRIIIa binding [6]		

**2**	**Homsap**	**G1v7**	**CH2**<br>**D3,**<br>**E117**	CH2<br>S3>D (239), I117>E (332)	S239D, I332E<br>DE	IGHG1 CH2<br>1.6-3 (231-239)<br>APELLGGPS ><br>APELLGGPD<br>FG 105-117 (322-332)<br>KVSNKA..LPAPI ><br>KVSNKA..LPAPE	**ADCC enhancement**. Increases FcγRIIIA binding [7].		
**2, 5**	**Homsap**	**G1v8**	**CH2**<br>**D3,**<br>**L115,**<br>**E117**	CH2<br>S3>D (239), A115>L (330), I117>E (332)	S239D, A330L, I332E<br>DLE, 3M	IGHG1 CH2<br>1.6-3 (231-239)<br>APELLGGPS ><br>APELLGGPD<br>FG 105-117 (322-332)<br>KVSNKA..LPAPI ><br>KVSNKA..LPLPE	**ADCC enhancement**. Increases FcRIIIA binding [7]<br>Decreases FcγRIIB binding [7]	**CDC reduction**. Ablates CDC [7]	3D [8]
**2**	**Homsap**	**G1v9**	**CH2**<br>**L7,**<br>**P83,**<br>**L85.2,**<br>**I88.**<br><br>**CH3**<br>**L83**	CH2<br>F7>L (243),<br>R83>P (292),<br>Y85.2>L (300),<br>V88>I (305).<br><br>CH3<br>P83>L (396)	F243L,<br>R292P,<br>Y300L,<br>V305I.<br><br>P396l<br>LPLIL	IGHG1 CH2<br>6-10 (242-246)<br>LFPPK ><br>LLPPK<br>83-88 (292-305)<br>REEQYNSTYRVVSV ><br>PEEQYNSTLRVVSI<br>CH3 83-84.4 (396-401)<br>PVLDSD ><br>IVLDSD	**ADCC enhancement**. 100% increase. [9]		
**2**	**Homsap**	**G1v10**	**CH2**<br>**Y1.3,**<br>**Q1.2,**<br>**W1.1,**<br>**M3,**<br>**D30,**<br>**E34,**<br>**A85.4**	CH2<br>L1.3>Y (234), L1.2>Q (235), G1.1>W (236), S3>M (239), H30>D (268), D34>E (270), S85.4>A (298)	L234Y,<br>L235Q,<br>G236W,<br>S239M,<br>H268D,<br>D270E,<br>S298A	IGHG1 CH2<br>1.6-3 (231-239)<br>APELLGGPS ><br>APEYQWGPM<br>27-31,34 (265-270)<br>DVSHED ><br>DVSDEE<br>84.1-85.1 (294-301)<br>EQYNSTYR ><br>EQYNATYR	**ADCC enhancement**. Increases FcγIIIA binding [10] >2000-fold (F158), >1000-fold (V158) in the association of G1v10 and G1v11 [10]		

**2**	**Homsap**	**G1v11**	**CH2**<br>**E34,**<br>**D109, M115,**<br>**E119**	CH2<br>D34>E (270), K109>D (326), A115>M (330)<br>K119>E (334)	D270E,<br>K326D,<br>A330M,<br>K334E	IGHG1 CH2<br>27-31,34 (265-270)<br>DVSHED ><br>DVSHEE<br>FG 105-117,118,119 (322-334)<br>KVSNKA..LPAPIEK ><br>KVSNDA..LPMPIEE	**ADCC enhancement**. Increases FcγIIIA binding [10] >2000-fold (F158), >1000-fold (V158) in the association of G1v10 and G1v11 [10]		
**3**	**Homsap**	**G1v12**	**CH2**<br>**A1.1,**<br>**D3,**<br>**L115,**<br>**E117**	CH2<br>G1.1>A (236),<br>S3>D (239), A115>L (330),<br>I117>E (332)	G236A,<br>S239D,<br>A330L,<br>I332E<br>GASDALIE	IGHG1 CH2<br>1.6-3 (231-239)<br>APELLGGPS ><br>APELLAGPD<br>FG 105-117 (322-332)<br>KVSNKA..LPAPI ><br>KVSNKA..LPLPE	**ADCC enhancement.** Increases FcγRIIIA binding [11].	**ADCP enhancement**. NK cell activation. Increases FcγRIIA binding [11]	5d4q,<br>5d6d
**3**	**Homsap**	**G1v13**	**CH2**<br>**A1.1,**<br>**D3,**<br>**E117**	CH2<br>G1.1>A (236),<br>S3>D (239),<br>I117>E (332)	G236A,<br>S239D,<br>I332E<br>GASDIE,<br>ADE	IGHG1 CH2<br>1.6-3 (231-239)<br>APELLGGPS ><br>APELLAGPD<br>FG 105-117 (322-332)<br>KVSNKA..LPAPI ><br>KVSNKA..LPAPE	**ADCC enhancement**. Increases FcγIIIA binding [12].	**ADCP enhancement**. NK cell activation. Increases FcγRIIA binding (70>fold). Increases FcγRIIA/FcγRIIB binding ratio (15-fold) [12]	
**6**	**Homsap**	**G1v14**	**CH2**<br>**A1.3,**<br>**A1.2**	CH2<br>L1.3>A (234), L1.2>A (235)	L234A,<br>L235A<br>LALA	IGHG1 CH2<br>1.6-3 (231-239)<br>APELLGGPS ><br>APEAAGGPS	**ADCC reduction**. Reduces FcγR binding [13,14].	**CDC reduction**. Reduces C1q binding [13,14]	
**6**	**Homsap**	**G1v14-1**	**CH2**<br>**A1.3,**<br>**A1.2,**<br>**A1**	CH2<br>L1.3>A (234), L1.2>A (235), G1>A (237)	L234A,<br>L235A,<br>G237A	IGHG1 CH2<br>1.6-3 (231-239)<br>APELLGGPS ><br>APEAAGAPS	**ADCC reduction**. Reduces FcγR binding.	**CDC reduction**. Reduces C1q binding.	Combines Homsap G1v14 and G1 CH2 A1.

**6**	**Homsap**	**G1v14-4**	**CH2**<br>**A1.3,**<br>**A1.2,**<br>**A114**	CH2<br>**L**1.3>**A** (234), **L**1.2>**A** (235, **P**114>**A** (329)	L234A,<br>L235A,<br>P329A	IGHG1 CH2<br>1.6-3 (231-239)<br>APELLGGPS ><br>APEAAGGPS<br>FG 105-117 (322-332)<br>KVSNKA..LPAPI ><br>KVSNKA..LAAPI	**ADCC reduction**. Reduces FcγR binding.	**CDC reduction**. Reduces C1q binding.	Combines Homsap G1v14 and Gv4.(G1 CH2 A114).
**6**	**Homsap**	**G1v14-48**	**CH2**<br>**A1.3,**<br>**A1.2,**<br>**R113**	CH2<br>**L**1.3>**A** (234), **L**1.2>**A** (235), **L**113>**R** (328)	L234A,<br>L235A,<br>L328R	IGHG1 CH2<br>1.6-3 (231-239)<br>APELLGGPS ><br>APEAAGGPS<br>FG 105-117 (322-332)<br>KVSNKA..LPAPI ><br>KVSNKA..RPAPI	**ADCC reduction**. Reduces FcγR binding.	**CDC reduction**. Reduces C1q binding.	Combines Homsap G1v14 and G1v48.(G1 CH2 R113).
**6**	**Homsap**	**G1v14-49**	**CH2**<br>**A1.3,**<br>**A1.2,**<br>**G114**	CH2<br>**L**1.3>**A** (234), **L**1.2>**A** (235), **P**114>**G** (329)	L234A,<br>L235A,<br>P329G<br>LALAPG	IGHG1 CH2<br>1.6-3 (231-239)<br>APELLGGPS ><br>APEAAGGPS<br>FG 105-117 (322-332)<br>KVSNKA..LPAPI ><br>KVSNKA..LGAPI	**ADCC reduction**. Reduces FcγR binding [15].	**CDC reduction**. Reduces C1q binding [15].	Combines Homsap G1v14 and G1v49 (G1 CH2 G114).
**6**	**Homsap**	**G1v14-67**	**CH2**<br>**A1.3,**<br>**A1.2,**<br>**S27**	CH2<br>**L**1.3>**A** (234), **L**1.2>**A** (235),<br>**D**27>**S** (265)	L234A,<br>L235A,<br>D265S	IGHG1 CH2<br>1.6-3 (231-239)<br>APELLGGPS ><br>APEAAGGPS<br>23-31 (261-269)<br>CVVVDVSHE ><br>CVVVSVSHE	**ADCC reduction**. Reduces FcγR binding [15].	**CDC reduction**. Reduces C1q binding [15].	Combines Homsap G1v14 and G1v67 (G1 CH2 S27).
**4**	**Homsap**	**G1v15**	CH2<br>**S118**	CH2<br>**E**118>**S** (333)	E333S	IGHG1 CH2<br>FG 105-117,118 (322-333)<br>KVSNKA..LPAPIE ><br>KVSNKA..LPAPIS	**CDC enhancement**. Increases C1q binding [5].		

**4**	**Homsap**	**G1v16**	CH2<br>**W109,**<br>**S118**	CH2<br>**K**109>**W** (326),<br>**E**118>**S** (333)	K326W,<br>E333S	IGHG1 CH2<br>FG 105-117,118 (322-333)<br>KVSNKA..LPAPIE ><br>KVSNWA..LPAPIS	**CDC enhancement**.<br>Increases C1q binding [5].		
**4**	**Homsap**	**G1v17**	CH2<br>**E29,**<br>**F30,**<br>**T107**	CH2<br>**S**29>**E** (267), **H**30>**F** (268), **S**107>**T** (324)	S267E, H268F, S324T<br>EFT	IGHG1 CH2<br>27-31 (265-269)<br>DVSHE ><br>DVEFE<br>FG 105-117 (322-332)<br>KVSNKA..LPAPI ><br>KVTNKA..LPAPI	**CDC enhancement**<br>Increases C1q binding [16].		
**4**	**Homsap**	**G1v18**	CH3<br>**R1,**<br>**G109,**<br>**Y120**	CH3<br>**E**1>**R** (345),<br>**E**109>**G** (430),<br>**S**120>**Y** (440)	E345R,<br>E430G,<br>S440Y	IGHG1 CH3<br>1.4-2 (341-346)<br>GQPREP ><br>GQPRRP<br>105-110 (426-431)<br>SVMHEA ><br>SVMHGA<br>118-125 (438-445)<br>QKSLSLSP ><br>QKYLSLSP	**CDC enhancement**.<br>Increases C1q binding [17]. The triple mutant IgG1-005-RGY (IGHG1v18) form IgG1 hexamers [17].	Favors IgG1 hexamerization.	
**5**	**Homsap**	**G1v19**	CH2<br>**A34**	CH2<br>**D**34>**A** (270)	D270A	IGHG1 CH2<br>34-41 (270-277)<br>DPEVKFNW ><br>APEVKFNW	**CDC reduction**. Reduces C1q binding [4]		
**5**	**Homsap**	**G1v20**	**CH2**<br>**A105**	CH2<br>**K**105>**A** (322)	K322A	IGHG1 CH2<br>FG 105-117 (322-332)<br>KVSNKA..LAPI ><br>AVSNKA..LPAPI	**CDC reduction**. Reduces C1q binding [4,14].		
**9**	**Homsap**	**G1v21**	**CH2**<br>**Y15.1,**<br>**T16,**<br>**E18**	CH2<br>**M**15.1>**Y** (252),<br>**S**16>**T** (254),<br>**T**18>**E** (256)	M252Y,<br>S254T,<br>T256E<br>YTE	IGHG1 CH2<br>13-18 (249-256)<br>DTLMISRT ><br>DTLYITRE	**Half-life increase**<br>Enhances FCGRT binding at pH 6.0 [18,19] (1).		
**9**	**Homsap**	**G1v22**	**CH2**	CH2		IGHG1 CH2	**Half-life increase**		

			**Y15.1,**<br>**T16,**<br>**E18.**<br>**CH3**<br>**K113,**<br>**F114,**<br>**H116**	**M**15.1>**Y** (252)<br>**S**16>**T** (254)<br>**T**18>**E** (256).<br>CH3<br>**H**113>**K** (433)<br>**N**114>**F** (434)<br>**Y**116>**H** (436)	M252Y<br>S254T<br>T256E.<br><br>H433K<br>N434F<br>Y436H	13-18 (249-256)<br>DTLMISRT ><br>DTLYITRE<br>CH3<br>FG 105-117 (426-437)<br>SVMHEA.LHNHYT ><br>SVMHEA.LKFHHT	Enhances FCGRT binding at pH 6.0 [19]		
**6**	**Homsap**	**G1v23**	**CH2**<br>**E1.2**	CH2<br>**L**1.2>**E** (235)	L235E	IGHG1 CH2<br>1.6-3 (231-239)<br>APELLGGPS ><br>APELEGGPS	**ADCC reduction**. Reduces FcγR binding [20]	**CDC reduction**. Reduces C1q binding [20]	
**9**	**Homsap**	**G1v24**	**CH3**<br>**L107,**<br>**S114**	CH3<br>**M**107>**L** (428),<br>**N**114>**S** (434)	M428L,<br>N434S	GHG1 CH3<br>FG 105-117 (426-437)<br>SVMHEA.LHNHYT ><br>SVLHEA.LHSHYT	**Half-life increase** Enhances FCGRT binding at pH 6.0 (11-fold increase in affinity) [21] (2).		
**7**	**Homsap**	**G1v25**	CH2<br>**E29,**<br>**F113**	CH2<br>**S**29>**E** (267), **L**113>**F** (328)	S267E,<br>L328F	IGHG1 CH2<br>27-31 (265-269)<br>DVSHE ><br>DVEHE<br>FG 105-117 (322-332)<br>KVSNKA..LPAPI **>**<br>KVSNKA..FPAPI	Increases FcγRIIB binding (400-fold) [22] Inhibits by downstream ITIM signaling in B cells [23]		
**14**	**Homsap**	**G1v26**	**CH3**<br>**Y22**	CH3<br>**T**22>**Y** (366)	T366Y	IGHG1 CH3<br>20-26 (364-370)<br>SLTCLVK ><br>SLYCLVK	**Knob** of knobs-into-holes G1v26 knob/G1v31 hole interactions between the CH3 of the two different gamma1 chains [24]		
**16**	**Homsap**	**G1v27**	**CH2**<br>**C3**	CH2<br>**S**3>**C** (329)	S239C	IGHG1 CH2<br>1.6-4 (231-240)<br>APELLGGPSV ><br>APELLGGPCV	Site-specific drug attachment engineered cysteine		
**16**	**Homsap**	**G1v28**	**CH2**<br>**C(3^4)**	CH2<br>(3^4)**C**(239^240)	**C**(239^240)	IGHG1 CH2<br>1.6-4 (231-240)<br>APELLGGPSV ><br>APELLGGPSCV	Site-specific drug attachment engineered cysteine		
**8**	**Homsap**	**G1v29**	**CH2**<br>**A84.4**	CH2<br>**N**84.4>**A** (297)	N297A	IGHG1 CH2<br>83-86<br>REEQYNSTYRVV ><br>REEQYASTYRVV	**ADCC reduction**. Reduces FcγR binding [25].	Owing to the absence of N-glycosylation at CH2 84.4 (297).	

**8**	**Homsap**	**G1v30**	**CH2**<br>**G84.4**	CH2<br>**N**84.4>**G** (297)	N297G	IGHG1 CH2<br>83-86<br>REEQYNSTYRVV ><br>REEQYGSTYRVV	**ADCC reduction**. Reduces FcγR binding [25].	Owing to the absence of N-glycosylation at CH2 84.4 (297).	
**14**	**Homsap**	**G1v31**	**CH3**<br>**T86**	CH3<br>**Y**86>**T** (407)	Y407T	IGHG1 CH3<br>85.4-89 (404-410)<br>GSFFLYSKL > GSFFLTSKL	**Hole** of knobs-into-holes G1v26 knob/G1v31 hole interactions between the CH3 of the two different gamma1 chains [24]<br>(G1v26 knob/G1v31 hole).		
**14**	**Homsap**	**G1v32**	**CH3**<br>**W22**	CH3<br>**T**22>**W** (366)	T366W	IGHG1 CH3<br>20-26 (364-370)<br>SLTCLVK ><br>SLWCLVK	**Knob** of knobs-into-holes G1v32 knob/G1v33 hole interactions between the CH3 of the two different gamma1 chains.		
**14**	**Homsap**	**G1v33**	**CH3**<br>**S22,**<br>**A24,**<br>**V86**	CH3<br>**T**22>**S** (366), **L**24>**A** (368), **Y**86>**V** (407)	T366S, L368A, Y407V	IGHG1 CH3<br>20-26 (364-370)<br>SLTCLVK ><br>SLSCAVK<br>85.4-89 (404-410)<br>GSFFLYSKL > GSFFLVSKL	**Hole** of knobs-into-holes G1v32 knob/G1v33 hole interactions between the CH3 of the two different gamma1 chains.		
**13**	**Homsap**	**G1v34**	CH3<br>**G109**	CH3<br>**E**109>**G** (430)	E430G	IGHG1 CH3<br>FG 105-117 (426-437) SVM-HEA.LHNHYT ><br>SVMHGA.LHNHYT	Favors IgG1 hexamerisation by increased intermolecular Fc-Fc interactions after antigen binding on the cell surface.		
**4**	**Homsap**	**G1v35**	**CH2**<br>**E29**	CH2<br>**S**29>**E** (267)	S267E<br>SE	IGHG1 CH2<br>27-31 (265-269)<br>DVSHE ><br>DVEHE	**CDC enhancement**. Increases C1q binding [16]	Binds to FCGRT and FcγRIIB,<br>but not to other FcγR in a mouse model [26]	
**8**	**Homsap**	**G1v36**	**CH2**<br>**Q84.4**	CH2<br>**N**84.4>**Q** (297)	N297Q	IGHG1 CH2<br>83-86<br>REEQYNSTYRVV ><br>REEQYQSTYRVV	**ADCC reduction**. Reduces FcγR binding	Owing to the absence of N-glycosylation at CH2 84.4 (297).	
**15**	**Homsap**	**G1v37**	**h**<br>**S5**	h<br>**C**5>**S** (220)	C220S	IGHG1 h<br>1-15 (216-230)<br>EPKSCDKTHTCPPCP ><br>EPKSSDKTHTCPPCP	No disulfide bridge inter H-L		

**6**	**Homsap**	**G1v38**	**CH2**<br>**S108,**<br>**F113**	CH2<br>**N**108>**S** (325), **L**113>**F** (328)	N325S, L328F	IGHG1 CH2<br>FG 105-117 (322-332)<br>KVSNKA..LPAPI ><br>KVSSKA..FPAPI	**ADCC reduction**.<br>Abrogates FcγRIII binding, increases FcγRII binding, retains FcγRI high affinity binding [27]	**CDC reduction**.<br>Abrogates C1q binding.	
**6**	**Homsap**	**G1v39**	**CH2**<br>**F1.3,**<br>**E1.2,**<br>**S116**	CH2<br>**L**1.3>**F** (234), **L**1.2>**E** (235), **P**116>**S** (331)	L234F,<br>L235E,<br>P331S<br>FES, TM	IGHG1 CH2<br>1.6-3 (231-239)<br>APELLGGPS ><br>APEFEGGPS<br>FG 105-117 (322-332)<br>KVSNKA..LPAPI ><br>KVSNKA..LPASI	**ADCC reduction**.<br>Reduces FcγR effector properties [28]	**CDC reduction**.<br>Reduces C1q binding [20]	3D<br>3c2s
**6**	**Homsap**	**G1v40**	**CH2**<br>**A1.3,**<br>**A1.2,**<br>**S116**	CH2<br>**L**1.3>**A** (234), **L**1.2>**A** (235),<br>**P**116>**S** (331)	L234A,<br>L235A,<br>P331S	IGHG1 CH2<br>1.6-3 (231-239)<br>APELLGGPS ><br>APEAAGGPS<br>FG 105-117 (322-332)<br>KVSNKA..LPAPI ><br>KVSNKA..LPASI<br>Homsap IGHG1v40 CH2 A1.3, A1.2, S116	**ADCC reduction**.<br>Reduces FcγR binding.	**CDC reduction**. Reduces C1q binding.	
**6**	**Homsap**	**G1v41**	**CH2**<br>**F1.3,**<br>**E1.2**	CH2<br>**L**1.3>**F** (234), **L**1.2>**E** (235)<br>FE	L234F, L235E	IGHG1 CH2<br>1.6-3 (231-239)<br>APELLGGPS ><br>APEFEGGPS	**ADCC reduction**.<br>Reduces FcγR binding [28]	**CDC reduction**. Reduces C1q binding [20]	
**9**	**Homsap**	**G1v42**	**CH2**<br>**Q14,**<br>**CH3**<br>**L107**	CH2<br>T14>Q (250)<br>CH3<br>M107>L (428)	T250Q<br><br>M428L	IGHG1 CH2<br>13-18 (249-256)<br>DTLMISRT ><br>DQLMISRT<br>CH3<br>FG 105-117 (426-437)<br>SVMHEA.LHNHYT ><br>SVLHEA.LHNHYT >	**Half-life increase**<br>Enhances FCGRT binding at pH 6.0 [19]		
**6**	**Homsap**	**G1v43**	**CH2**<br>**A1.3,**<br>**E1.2,**<br>**A1**	CH2<br>**L**1.3>**A** (234), **L**1.2>**E** (235), **G**1>**A** (237)	L234A,<br>L235E,<br>G237A	IGHG1 CH2<br>1.6-3 (231-239)<br>APELLGGPS ><br>APEAEGAPS	**ADCC reduction**. Reduces FcγR binding.	**CDC reduction**. Reduces C1q binding.	
**16**	**Homsap**	**G1v44**	**CH3**<br>**C122**	CH3<br>**S**122>**C** (442)	S442C	IGHG1 CH3<br>118-125 (438-445)<br>QKSLSLSP >	Conjugation site-specific engineered cysteine		

						QKSLCLSP			
3	**Homsap**	**G1v45**	**CH2**<br>**A1.1,**<br>**L115,**<br>**E117**	CH2<br>G1.1>A (236), A115>L (330), I117>E (332)	G236A,<br>A330L,<br>I332E<br>GAALIE	IGHG1 CH2<br>1.6-3 (231-239)<br>APELLGGPS ><br>APELLAGPS<br>FG 105-117 (322-332)<br>KVSNKA..LPAPI ><br>KVSNKA..LPLPE	**ADCC enhancement**. Increases FcγIIIA binding.	**ADCP enhancement**. NK cell activation.	
9	**Homsap**	**G1v46**	**CH3**<br>**K113,**<br>**F114**	CH3<br>H113>K (433),<br>N114>F (434)	H433K,<br>N434F	IGHG1 CH3<br>FG 105-117 (426-437) SVM-HEA.LHNHYT > SVM-HEA.LKFHYT	**Half-life increase**<br>Enhances FCGRT binding at pH 6.0.		
1	**Homsap**	**G1v47**	**CH2**<br>**delG1.1**	CH2<br>G1.1>del (326)	G236del	IGHG1 CH2<br>1.6-3 (231-239)<br>APELLGGPS ><br>APELL - GPS	**ADCC reduction**. Eliminates binding to FcγRI, FcγRIIA, FcγRIIIA [29].		
6	**Homsap**	**G1v48**	**CH2**<br>**R113**	CH2<br>L113>R (328)	L328R	IGHG1 CH2<br>FG 105-117 (322-332)<br>KVSNKA..LPAPI ><br>KVSNKA..RPAPI	**ADCC reduction**. Reduces FcγR binding.	**CDC reduction**. Reduces C1q binding.	
6	**Homsap**	**G1v49**	**CH2**<br>**G114**	CH2<br>P114>G (329)	P329G	IGHG1 CH2<br>FG 105-117 (322-332)<br>KVSNKA..LPAPI ><br>KVSNKA..LGAPI	**ADCC reduction**. Reduces FcγR binding [15].	**CDC reduction**. Reduces C1q binding [15].	
1	**Homsap**	**G1v50**	**CH2**<br>**P1.4,**<br>**V1.3,**<br>**A1.2,**<br>**delG1.1**	CH2<br>E1.4>P (233), L1.3>V (234), L1.2>A (235), G1.1>del (236)	E233P,<br>L234V,<br>L235A,<br>G236del	IGHG1 CH2<br>1.6-3 (231-239)<br>APELLGGPS ><br>APPVA-GPS	**ADCC reduction**. Decreases FcgammaR binding (G2-like motif combines Homsap G1v1, v2, v3 and v47].		
6	**Homsap**	**G1v51**	**CH2**<br>**K29**	CH2<br>S29>K (267)	S267K	IGHG1 CH2<br>27-31 (265-269)<br>DVSHE ><br>DVKHE	**ADCC reduction**. Reduces FcγR binding.	**CDC reduction**. Reduces C1q binding.	
1	**Homsap**	**G1v52**	**CH2**<br>**R1.1,**<br>**R113**	G1.1>R (231)<br>L113>R (328)	G236R,<br>L328R<br>GRLR	IGHG1 CH2<br>1.6-3 (231-239)<br>APELLGGPS ><br>APELLRGPS	**ADCC reduction**. Abrogates FcgammaR binding.		

						IGHG1 CH2<br>FG 105-117 (322-332)<br>KVSNKA..LPAPI ><br>KVSNKA..RPAPI			
**6**	**Homsap**	**G1v53**	**CH2**<br>**F1.3,**<br>**Q1.2,**<br>**Q105**	CH2<br>**L**1.3>**F** (234)<br>**L**1.2>**Q** (235)<br>**K**105> **Q** (322)	L234F,<br>L235Q,<br>K322Q,<br>FQQ	IGHG1 CH2<br>1.6-3 (231-239)<br>APELLGGPS ><br>APEFQGGPS<br>FG 105-117 (322-332)<br>KVSNKA..LPAPI ><br>QVSNKA..LPAPI	**ADCC reduction**. Reduces FcγR binding.	**CDC reduction**. Reduces C1q binding.	
**11**	**Homsap**	**G1v54**	**CH2**<br>**C83,**<br>**C85**	CH2<br>**R**83>**C** (292), **V**85>**C** (302)	R292C,<br>V302C	IGHG1 CH2<br>83-86<br>REEQYNSTYRVV ><br>CEEQYASTYR[illegible]V (v29)<br>CEEQYGSTYRCV (v30)<br>CEEQYQSTYRCV (v36)	Stabilizes CH2 in the absence of N84.4 (297) glycosylation (e.g. with G1v29, G1v30 or G1v36).		
**11, 8**	**Homsap**	**G1v54-29**	**CH2**<br>**C83,**<br>**A84.4,**<br>**C85**	CH2<br>**R**83>**C** (292),<br>**N**84.4>**A**(297)<br>**V**85>**C** (302)	R292C,<br>N297A<br>V302C	IGHG1 CH2<br>83-86 (292-303)<br>REEQYNSTYRVV ><br>CEEQYASTYRCV	Stabilizes CH2 in the absence of N84.4 (297) glycosylation (Gv1-54).	**ADCC reduction**. Reduces FcγR binding [25] (G1v29).	Combines Homsap G1v54 and G1v29.
**11, 8**	**Homsap**	**G1v54-30**	**CH2**<br>**C83,**<br>**G84.4,**<br>**C85**	CH2<br>**R**83>**C** (292),<br>**N**84.4>**G** (297)<br>**V**85>**C** (302)	R292C,<br>N297G<br>V302C	IGHG1 CH2<br>83-86 (292-303)<br>REEQYNSTYRVV ><br>CEEQYGSTYRCV	Stabilizes CH2 in the absence of N84.4 (297) glycosylation (G1v54)	**ADCC reduction**. Reduces FcγR binding [25] (Gv1-30).	Combines Homsap G1v54 and G1v30.
**11, 8**	**Homsap**	**G1v54-36**	**CH2**<br>**C83,**<br>**Q84.4,**<br>**C85**	CH2<br>**R**83>**C** (292),<br>**N**84.4>**Q** (297)<br>**V**85>**C** (302)	R292C,<br>N297Q<br>V302C	IGHG1 CH2<br>83-86 (292-303)<br>REEQYNSTYRVV ><br>[illegible]EEQY[illegible]STYR[illegible]V	Stabilizes CH2 in the absence of N84.4 (297) glycosylation (G1v54).	**ADCC reduction**. Reduces FcγR binding [25] (Gv1-36).	Combines Homsap G1v54 and G1v36.
**16**	**Homsap**	**G1v55**	**CH3**<br>**C123**	CH3<br>**L**123>**C** (443)	L443C	IGHG1 CH3<br>118-125 (438-445)<br>QKSLSLSP ><br>QKSLSCSP	Conjugation site-specific engineered cysteine		
**16**	**Homsap**	**G1v56**	**CH2**<br>**F85.2**<br>**CH3**<br>**F85.2**	CH2<br>**Y**85.2>**F** (pAMF)<br>CH3<br>**F**85.2>**F** (pAMF)	Y300F<br><br><br>F404F	IGHG1 CH2<br>84.1-85.1 (294-301)<br>EQYNSTYR ><br>EQYNSTFR<br>CH3<br>84.1-85.1 (398-405)<br>LDSDGSFF<br>LDSDGSFF	Modified phenylalanine for conjugation (produced in *Escherichia coli*, non glycosylated)		
**17**	**Homsap**	**G1v57**	**CH1**<br>**E26,**	CH1	K147E, K213E	IGHG1 CH1<br>23-26 (144-147)	Enhances, with KCv57, the hetero pairing H-L of bispecific antibodies		

			**E119**	K26>E (147), K119>E (213)		CLVK ><br>CLVE<br>118-121 (212-215)<br>DKKV ><br>DEKV			
**17**	**Homsap**	**KCv57**	**IGKC**<br>**R12,**<br>**K13**	IGKC<br>E12>R,<br>Q13>K	E123R, Q124K	IGKC<br>10-15 (121-126)<br>SDEQLK ><br>SDRKLK	Enhances, with G1v57, the hetero pairing H-L of bispecific antibodies		
**17**	**Homsap**	**G1v58**	**CH1**<br>**C5,**<br>**h**<br>**V5**	CH1<br>F5>C (126),<br>h<br>C5>V (220)	F126C,<br><br>C220V	IGHG1 CH1<br>1.4-15 (118-136)<br>ASTKGPSVFPLAPSSKSTS ><br>ASTKGPSVCPLAPSSKSTS<br>IGHG1 h<br>1-15 (216-230)<br>EPKSCDKTHTCPPCP ><br>EPKSVDKTHTCPPCP	Alternative interchain cysteine mutations to enhance, with LC2v58, the heteropairing H-L of bispecific antibodies		
**17**	**Homsap**	**LC2v58**	**LC2**<br>**C10,**<br>**V126**	IGLC<br>S10>C (121), C126>V (214)	S121C,<br>C214V	IGLC2<br>1.5-15 (107A-126)<br>GQPKAAPSVTLFPPSSEELQ ><br>GQPKAAPSVTLFPPCSEELQ<br>IGLC2<br>118-127 (206-215)<br>EKTVAPTECS ><br>EKTVAPTEVS	Alternative interchain cysteine mutations to enhance, with G1v58, the heteropairing H-L of bispecific antibodies		
6	**Homsap**	G1v59	**CH2**<br>**S1.3**<br>**T1.2**<br>**R1.1**	CH2<br>L1.3>S (234)<br>L1.2>T (235)<br>G1.1>R (236)	L234S<br>L235T<br>G236R	IGHG1 CH2<br>1.6-3 (231-239)<br>APELLGGPS > APESTRGPS	**ADCC undetectable.** Abrogates FcγR binding [30].	**CDC undetectable.** Abrogates C1q binding [30].	
6	**Homsap**	G1v60	CH2<br>**S115,**<br>**S116**	CH2<br>A115>S (330)<br>P116>S (331)	A330S<br>P331S	FG 105-117 (322-332)<br>KVSNKA..LPAPI ><br>QVSNKA..LPSSI	**ADCC reduction**. Reduces FcγR binding	**CDC reduction**. Reduces C1q binding.	
**15**	**Homsap**	**G1v61**	**h**<br>**S11**	h<br>C11>S (226)	C226S	IGHG1 h<br>1-15 (216-230)<br>EPKSCDKTHTCPPCP ><br>EPKSCDKTHTSPPCP	No disulfide bridge inter H-H h 11.		.

**15**	**Homsap**	**G1v62**	**h**<br>**S14**	h<br>C14>S (229)	C229S	IGHG1 h<br>1-15 (216-230)<br>EPKSCDKTHTCPPCP ><br>EPKSCDKTHTCPPSP	No disulfide bridge inter H-H h 14.		
**6**	**Homsap**	**G1v63**	**CH2**<br>**S2**	CH2<br>P2>S	P238S	IGHG1 CH2<br>1.6-3 (231-239)<br>APELLGGPS ><br>APELLGGSS	**ADCC reduction**. Reduces FcγR binding.	**CDC reduction**. Reduces C1q binding.	
**16**	**Homsap**	**G1v64**	**CH2**<br>**C36**	CH2<br>E36>C	E272C	IGHG1 CH2<br>34-41 (270-277)<br>DPEVKFNW ><br>DPCVKFNW	Conjugation site-specific engineered cysteine		
**6**	**Homsap**	**G1v65**	**CH2**<br>**delE1.4,**<br>**delL1.3,**<br>**delL1.2**	CH2<br>E1.4>del,<br>L1.3>del,<br>L1.2>del	E233del,<br>L234del,<br>L235del	IGHG1 CH2<br>1.6-3 (231-239)<br>APELLGGPS ><br>AP- - - GGPS	**ADCC reduction**. Reduces FcγR binding.	**CDC reduction**. Reduces C1q binding.	
**1**	**Homsap**	**G1v66**	**CH2**<br>**A27**	CH2<br>D27>A	D265A	IGHG1 CH2<br>23-31 (261-269)<br>CVVVDVSHE ><br>CVVVAVSHE	**ADCC reduction**. Reduces FcγR binding.		
**1**	**Homsap**	**G1v67**	**CH2**<br>**S27**	CH2<br>D27>S	D265S	IGHG1 CH2<br>23-31 (261-269)<br>CVVVDVSHE ><br>CVVVSVSHE	**ADCC reduction**. Reduces FcγR binding.		
**14**	**Homsap**	**G1v68**	**CH3**<br>**V6,**<br>**L22,**<br>**L79,**<br>**W81**	CH3<br>T6>V (350)<br>T22>L (366)<br>K79>L (392)<br>T81>W (394)	T350V<br>T366L<br>K392L<br>T394W	IGHG1 CH3<br>3-9 (347-353)<br>QVYTLPP ><br>QVYVLPP<br>20-26 (364-370)<br>SLTCLVK ><br>SLLCLVK<br>77-83 (390-396)<br>NYKTTPP ><br>NYLTWPP	Enhances, with G1v69, the heteropairing H-H of bispecific antibodies		

**14**	**Homsap**	**G1v69**	**CH3**<br>**V6,**<br>**Y7,**<br>**A85.1,**<br>**V86**	CH3<br>**T**6>**V** (350)<br>**L**7>**Y** (351)<br>**F**85.1>**A** (405)<br>**Y**86>**V** (407)	T350V<br>L351Y<br>F405A<br>Y407V	IGHG1 CH3<br>3-9 (347-353)<br>QVYTLPP ><br>QVYVYPP<br>IGHG1 CH3<br>85.4-89 (404-410)<br>GSFFLYSKL ><br>GSFALVSKL	Enhances, with G1v68, the heteropairing H-H of bispecific antibodies		
**6**	**Homsap**	**G1v70**	**h**<br>**S5,**<br>**S11,**<br>**S14,**<br>**CH2**<br>**S2**	h<br>**C**5>**S** (220),<br>**C**11>**S** (226)<br>**C**14>**S** (226)<br>CH2<br>**P**2>**S**	C220S<br>C226S<br>C229S<br><br>P238S	IGHG1 h<br>1-15 (216-230)<br>EPKSCDKTHTCPPCP ><br>EPKSSDKTHTSPPSP<br>IGHG1 CH2<br>1.6-3 (231-239)<br>APELLGGPS ><br>APELLGGSS	**ADCC reduction**. Reduces FcγR binding.	**CDC reduction.** Reduces C1q binding.	Combines G1v63 with G1v37 (no H-L), G1v61 (no H-H h11) and G1v62 (no H-H h14).
**6, 9**	**Homsap**	**G1v53,**<br>**G1v21**	**CH2**<br>**F1.3,**<br>**Q1.2,**<br>**Q105**<br>**Y15.1,**<br>**T16,**<br>**E18**	CH2<br>**L**1.3>**F** (234),<br>**L**1.2>**Q** (235),<br>**K**105> **Q** (322)<br>**M**15.1>**Y** (252),<br>**S**16>**T** (254), **T**18>**E** (256)	L234F,<br>L235Q,<br>K322Q,<br>M252Y,<br>S254T,<br>T256E<br>FQQ-YTE	IGHG1 CH2<br>1.6-3 (231-239)<br>APELLGGPS ><br>APEFQGGPS<br>15-18 (251-256)<br>LMI.SRT ><br>LYITRE<br>FG 105-117 (322-332)<br>KVSNKA..LPAPI ><br>QVSNKA..LPAPI	**ADCC reduction**. Reduces FcγR binding [31] (G1v53).	**CDC reduction**. Reduces C1q binding [31] (G1v53).	**Half-life increase**<br>Enhances FCGRT binding at pH 6.0 [32] [19] (G1v21)..
**4**	**Homsap**	**G1G3v1**	CH2<br>**Q**38,<br>**K**40,<br>**F**85.2	CH2<br>**K**38>**Q** (274),<br>**N**40>**K** (276),<br>**Y**85.2>**F** (300)	K274Q,<br>N276K,<br>Y300F<br>chimere G1-G3 (1)	IGHG1 CH2<br>34-41 (270-277)<br>DPEVKFNW ><br>DPEVQFKW<br>84.1-85.1 (294-301)<br>EQYNSTYR ><br>EQYNSTFR	**CDC enhancement**. Increases C1q binding [33].		
**2**	**Homsap**	**G2v1**	**CH2**<br>**L1.3,**<br>**L1.2,**<br>**G1.1**	CH2<br>**V1.2**>**LL** (234,235)<br>**A**1.1>**G** (236)	V235LL,<br>A236G	IGHG2 CH2<br>1.6-3 (231-239)<br>AP.PVAGPS ><br>APPLLGGPS	**ADCC enhancement**. Confers FcγRI binding (WT does not show any binding capacity) [3]		

**6**	**Homsap**	**G2v2**	**CH2**<br>**Q30,**<br>**L92,**<br>**S115,**<br>**S116**	CH2<br>**H**30>**Q** (268),<br>**V**92>**L** (309),<br>**A**115>**S** (330),<br>**P**116>**S** (331)	H268Q,<br>V309L,<br>A330S,<br>P331S<br>IgG2m4	IGHG2 CH2<br>27-38 (265-274)<br>DVSHEDPEVQ ><br>DVSQEDPEVQ<br>89-96 (306-313)<br>LTVVHQDW ><br>LTVLHQDW<br>FG 105-117 (322-332)<br>KVSNKG..LPAPI ><br>KVSNKA..LPSSI	**ADCC reduction**. Reduces FcγR binding [34]	**CDC reduction**. Reduces C1q binding [34]	
**6**	**Homsap**	**G2v3**	**CH2**<br>**A1.2,**<br>**A1,**<br>**S2,**<br>**A30,**<br>**L92,**<br>**S115,**<br>**S116**	CH2<br>**V**1.2>**A** (235),<br>**G**1>**A** (237),<br>**P**2>**S** (238),<br>**H**30>**A** (268),<br>**V**92>**L** (309),<br>**A**115>**S** (330),<br>**P**116>**S** (331)	V235A,<br>G237A,<br>P238S,<br>H268A,<br>V309L,<br>A330S,<br>P331S<br>G2sigma	IGHG2 CH2<br>1.6-3 (231-239)<br>AP.PVAGPS ><br>AP.PAAASS<br>27-38 (265-274)<br>DVSHEDPEVQ ><br>DVSAEDPEVQ<br>89-96 (306-313)<br>LTVVHQDW ><br>LTVLHQDW<br>FG 105-117 (322-332)<br>KVSNKG..LPAPI ><br>KVSNKA..LPSSI	**ADCC reduction**. Reduces FcγR binding [28]. Undetectable ADCC andV1 ADCP [28]	**CDC reduction**. Reduces C1q binding [28]. Undetectable CDC [28]	
**9**	**Homsap**	**G2v4**	**CH2**<br>**Q14**	CH2<br>**T**14>**Q** (250)	T250Q	IGHG2 CH2<br>13-18 (249-256)<br>DTLMISRT ><br>DQLMISRT	**Half-life increase**<br>Enhances FCGRT binding at pH 6.0 [35]		
**9**	**Homsap**	**G2v5**	**CH3**<br>**L107**	CH3<br>**M**107>**L** (428)	M428L	IGHG2 CH3<br>FG 105-117 (426-437)<br>SVMHEA.LHNHYT ><br>SVLHEA.LHNHYT	**Half-life increase**<br>Enhances FCGRT binding at pH 6.0 [35]		
**9**	**Homsap**	**G2v6**	**CH2**<br>**Q14,**<br>**CH3**<br>**L107**	CH2<br>**T**14>**Q** (250)<br>CH3<br>**M**107>**L** (428)	T250Q<br><br>M428L	IGHG2 CH2<br>13-18 (249-256)<br>DTLMISRT ><br>DQLMISRT<br>CH3<br>FG 105-117 (426-437)<br>SVMHEA.LHNHYT ><br>SVLHEA.LHNHYT	**Half-life increase**<br>Enhances FCGRT binding at pH 6.0 [35]		

**19, 6**	**Homsap**	**G2v7**	**CH2**<br>**Y85.2,**<br>**L92,**<br>**A339**	CH2<br>**F**85.2>**Y** (300)<br>**V**92>**L** (309)<br>**T**339>**A** (339)	F300Y<br>V309L<br>T339A	IGHG2 CH2<br>85.4- 92 (300-309)<br>STFRVVSVLTVV ><br>STYRVVSVLTVL<br>118-125 (333-340)<br>EKTISKTK ><br>EKTISKAK	Reduces acid-induced aggregation [36]	**Low ADCC** Low FcγR binding [36]	**Low CDC**<br>Low C1q binding [36]
**9**	**Homsap**	**G2v8-1**	**CH2**<br>**A93**	CH2<br>**H**93>**A** (310)	H310A	IGHG2 CH2<br>89-96 (306-313)<br>LTVVHQDW ><br>LTVVAQDW	**Abrogates FCGRT binding** at pH 6.0 (G2v8 for any amino acid replacement of H93 except cystein) [37]. Number 1 of G2v8-1 is for A.		
**6**	**Homsap**	**G2G4v1 (1)**	**CH2**<br>**E1.4>del**<br>**P1.3,**<br>**V1.2,**<br>**A1.1**	CH2<br>**E**1.4>**del** (233),<br>**F**1.3>**P** (234),<br>**L**1.2>**V** (235),<br>**G**1.1>**A** (236)	E233delF234P,<br>L235V,<br>G236A	IGHG4 CH2<br>1.6-3 (231-239)<br>APEFLGGPS ><br>AP.PVAGPS	**ADCC reduction**. Reduces FcγR binding [38]	**CDC reduction**. Reduces C1q binding [38]	
**9**	**Homsap**	**G3v1**	**CH3**<br>**H115**	CH3<br>**R**115>**H** (435)	R435H	IGHG3 CH3<br>FG 105-117 (426-437)<br>SVMHEA.LHNRFT ><br>SVMHEA.LHNHFT	**Half-life increase**<br>Extends half-life [39]		
**2**	**Homsap**	**G4v1**	**CH2**<br>**L1.3**	CH2<br>**F**1.3>**L** (234)	F234L	IGHG4 CH2<br>1.6-3 (231-239)<br>APEFLGGPS ><br>APELLGGPS	**ADCC enhancement**. Increases FcγRI affinity [3].		
**4**	**Homsap**	**G4v2**	CH2<br>**P**116	CH2<br>**S**116>**P** (331)	S331P	IGHG4 CH2<br>FG 105-117 (322-332)<br>KVSNKG..LPSSI ><br>KVSNKG..LPSPI	**CDC enhancement** [40]<br>(G1-, G2-, G3-like).		
**6**	**Homsap**	**G4v3**	**CH2**<br>**E1.2**	CH2<br>**L**1.2>**E** (235)	L235E<br>LE	IGHG4 CH2<br>1.6-3 (231-239)<br>APEFLGGPS ><br>APEFEGGPS	**ADCC reduction**. Reduces FcγR binding [20]	**CDC reduction**. Reduces C1q binding [20]	

**6**	**Homsap**	**G4v3-49**	**CH2**<br>**E1.2**<br>**G114**	CH2<br>**L**1.2>**E** (235)<br>**P**114>**G** (329)	L235E<br>P329G<br>LEPG	IGHG4 CH2<br>1.6-3 (231-239)<br>APEFLGGPS ><br>APEFEGGPS<br>FG 105-117 (322-332)<br>KVSNKG..LPSSI ><br>KVSNKG..LGSSI	**ADCC reduction**. Reduces FcγR binding [15].	**CDC reduction**. Reduces C1q binding [15].	Combines G4v3 (G4 CH2 E1.2) and G4v49.(G4 CH2 G114).
**6**	**Homsap**	**G4v4**	**CH2**<br>**A1.3,**<br>**A1.2**	CH2<br>**F**1.3>**A** (234), **L**1.2>**A** (235)	F234A<br>L235A<br>FALA	IGHG4 CH2<br>1.6-3 (231-239)<br>APEFLGGPS ><br>APEAAGGPS	**ADCC reduction**. Reduces FcγR binding [13]	**CDC reduction**. Reduces C1q binding [13]	
**12**	**Homsap**	**G4v5**	**h**<br>**P10**	h<br>**S**10>**P** (228)	S228P	IGHG4 h<br>1-12 (216-230)<br>ESKYGPPCPSCP ><br>ESKYGPPCPPCP<br>(G1-like)	Prevents *in vivo* and *in vitro* IgG4 half-IG exchange [41]		
**12**	**Homsap**	**G4v6**	**CH3**<br>**K88**	**CH3**<br>**R**88>**K**	R409K	IGHG1 CH3<br>85.4-89 (404-410)<br>GSFFLYSRL ><br>GSFFLYSKL	Reduces IgG4 half-IG exchange [42]. IGHG4 CH3 K88 is an allelic polymorphism present in Homsap IGHG4*03 (CH3 codon 69) [43].		
**6**	**Homsap**	**G4v7**	**CH2**<br>**delE1.4**<br>**P1.3,**<br>**V1.2,**<br>**A1.1, -**	CH2<br>**E**1.4>del (233)<br>**F**1.3>**P** (234), **L**1.2>**V** (235), **G**1.1>**A** (236),	E233del,<br>F234P,<br>L235V,<br>G236A	IGHG4 CH2<br>1.6-3 (231-239)<br>APEFLGGPS><br>AP-PVAGPS<br>(G2-like)	**ADCC reduction**. Reduces FcγR binding.	**CDC reduction**. Reduces C1q binding.	
**10**	**Homsap**	**G4v8**	**CH3**<br>**R115,**<br>**F116,**<br>**P125**	CH3<br>**H**115>**R** (435), **Y**116>**F** (436), **L**125>**P** (445)	H435R,<br>Y436F, L445P	IGHG4 CH3-<br>FG 105-117 (426-437)<br>SVMHEA.LHNHYT ><br>SVMHEA.LHNRFT<br>118-125 (438-445)<br>QKSLSLSL ><br>QKSLSLSP	Abrogates binding to Protein A		
**18**	**Homsap**	**G4v10**	**CH3**<br>**L85.1,**<br>**K88**	CH3<br>**F**85.1>**L** (405), **R**88>**K** (409)	F405L,<br>R409K	IGHG1 CH3<br>85.4-92 (402-413)<br>GSFFLYSRLTVD ><br>GSFLLYSKLTVD	Control of half-IG exchange of bispecific IgG4		
**9**	**Homsap**	**G4v21**	**CH2**<br>**Y15.1,**<br>**T16,**	CH2<br>**M**15.1>**Y** (252),	M252Y,<br>S254T,	IGHG4 CH2<br>13-18 (249-256)<br>DTLMISRT >	**Half-life increase**<br>Enhances FCGRT binding at pH 6.0 [19].		

			E18	S16>T (254), T18>E (256)	T256E<br>YTE	DTLYITRE			
9	**Homsap**	**G4v22**	**CH2**<br>**T16,**<br>**P91,**<br>**CH3**<br>**A114**	CH2<br>S16>T (254),<br>V91>P (308)<br>CH3<br>N114>A (434)	S254T<br>V308P<br><br>N434A	IGHG4 CH2<br>13-18 (249-256)<br>DTLMISRT ><br>DTLYITRE<br>CH3<br>FG 105-117 (426-437)<br>SVMHEA.LHNHYT ><br>SVMHEA.LHAHYT	**Half-life increase**<br>Enhances FCGRT binding at pH 6.0 [44]		
9	**Homsap**	**G4v24**	**CH3**<br>**L107**<br>**S114**	CH3<br>M107>L (428)<br>N114>S (434)	M428LN434A	CH3<br>FG 105-117 (426-437)<br>SVMHEA.LHNHYT ><br>SVLHEA.LHSHYT	**Half-life increase**<br>Enhances FCGRT binding at pH 6.0.		
8	**Homsap**	**G4v36**	**CH2**<br>**Q84.4**	CH2<br>N84.4>Q (297)	N297Q	IGHG4 CH2<br>83-86<br>REEQFNSTYRVV ><br>REEQFQSTYRVV	**ADCC reduction**. Reduces FcγR binding	Owing to the absence of N-glycosylation at CH2 84.4 (297).	
6	**Homsap**	**G4v49**	**CH2**<br>**G114**	CH2 P114>G (329)	P329G	IGHG4 CH2<br>FG 105-117 (322-332)<br>KVSNKG..LPSSI ><br>KVSNKG..LGSSI	**ADCC reduction**. Reduces FcγR binding [15].	**CDC reduction**. Reduces C1q binding [15].	
6, 12	**Homsap**	**G4v3**<br>**G4v5**	**h**<br>**P10,**<br>**CH2**<br>**E1.2**	h<br>S10>P (228)<br>CH2<br>L1.2>E (235)	S228P,<br><br>L235E<br>SPLE	IGHG4 h<br>1-12 (216-230)<br>ESKYGPPCPSCP<br>ESKYGPPCPPCP<br>CH2<br>1.6-3 (231-239)<br>APEFLGGPS ><br>APEFEGGPS	**ADCC reduction**. Reduces FcγR binding [20] (G4v3).	**CDC reduction**. Reduces C1q binding [20] (G4v3).	Prevents *in vivo* and *in vitro* IgG4 half-IG exchange [41] (G4v5).
6, 12	**Homsap**	**G4v3-49**<br>**G4v5**	**h**<br>**P10,**<br>**CH2**<br>**E1.2**<br>**G114**	h<br>S10>P (228)<br>CH2<br>L1.2>E (235)<br>P114>G (329)	S228P,<br><br>L235E<br>P329G<br>SPLEPG	IGHG4 h<br>1-12 (216-230)<br>ESKYGPPCPSCP<br>ESKYGPPCPPCP<br>CH2<br>1.6-3 (231-239)<br>APEFLGGPS ><br>APEFEGGPS	**ADCC reduction**. Reduces FcγR binding [15] (G4v3-49).	**CDC reduction**. Reduces C1q binding [15] (G4v3-49).	Prevents *in vivo* and *in vitro* IgG4 half-IG exchange [41] (G4v5).

						FG 105-117 (322-332)<br>KVSNKG..LPSSI ><br>KVSNKA..LGSSI			
**6, 12**	**Homsap**	**G4v4**<br>**G4v5**	**h**<br>**P10,**<br>**CH2**<br>**A1.3,**<br>**A1.2**	h<br>**S**10>**P** (228)<br>CH2<br>**F**1.3>**A** (234)<br>**L**1.2>**A** (235)	S228P,<br><br>F234A,<br>L235A<br>IgG4 ProA-laAla	IGHG4 h<br>1-12 (216-230)<br>ESKYGPPCPSCP<br>ESKYGPPCPPCP<br>CH2<br>1.6-3 (231-239)<br>APEFLGGPS ><br>APEAAGGPS	**ADCC reduction**. Reduces FcγR binding [28] (G4v4).	**CDC reduction**. Reduces C1q binding [13] (G4v4)	Prevents *in vivo* and *in vitro* IgG4 half-IG exchange [41] (G4v5).
**6**	**Canlupfam**	**G2v1**	**CH2**<br>**A1.3,**<br>**A1.2,**<br>**A1**	CH2<br>**M**1.3>**A** (234), **L**1.2>**A** (235), **G**1>**A** (237).	M234A<br>L235A<br>G237A	IGHG2 CH2<br>1.6-3 (231-239)<br>APEMLGGPS ><br>APEAAGAPS	**ADCC reduction**. Reduces FcγR binding	**CDC reduction**. Reduces C1q binding	
**6**	**Canlupfam**	**G2v2**	**CH2**<br>**A1.3,**<br>**A1.2,**<br>**G114**	CH2<br>**M**1.3>**A** (234), **L**1.2>**A** (235)<br>**P**114>**G** (329)	M234A<br>L235A<br>P329G	IGHG2 CH2<br>1.6-3 (231-239)<br>APEMLGGPS ><br>APEAAGGPS<br>IGHG1 CH2<br>FG 105-117 (322-332)<br>KVNNKA..LPSPI ><br>KVNNKA..LGSPI	**ADCC reduction**. Reduces FcγR binding	**CDC reduction**. Reduces C1q binding	
**8**	**Canlupfam**	**G2v29**	**CH2**<br>**A84.4**	CH2<br>**N**84.4>**A** (297)	N297A	IGHG1 CH2<br>83-86<br>RE**EQFNGTYR**VV ><br>RE**EQFAGTYR**VV	**ADCC reduction**. Reduces FcγR binding		
**2**	**Musmus**	**G2Bv1**	**CH2**<br>**L1.2**	CH2<br>**E**1.2>**L** (235)	E235L	IGHG2B CH2<br>1.6-3 (231-239)<br>APNLEGGPS ><br>APNLLGGPS	**ADCC enhancement**. Increases FcγRI affinity [45]		
**5**	**Musmus**	**G2Bv2**	CH2<br>**A101**	CH2<br>**E**101>**A** (318)	E318A	IGHG2B CH2<br>100-110<br>KEFKCKVNNKD ><br>KAFKCKVNNKD	**CDC reduction**. Reduces C1q binding [46].		

**5**	**Musmus**	**G2Bv3**	CH2<br>**A103**	CH2<br>**K**103>**A** (320)	K320A	IGHG2B CH2<br>100-110<br>KEF**KC**KVNNKD ><br>KEF**AC**KVNNKD	**CDC reduction**. Reduces C1q binding [46]		
**5**	**Musmus**	**G2Bv4**	CH2<br>**A105**	CH2<br>**K**105>**A** (322)	K322A	IGHG2B CH2<br>100-110<br>KEFK**CK**VNNKD ><br>KEFK**CA**VNNKD	**CDC reduction**. Reduces C1q binding [46]		

Engineered amino acid changes are in bold in the IMGT variants (red before the change, green after the change. The motif is in yellow and shown before and after the AA change(s). Amino acids of the motifs at additional positions in the IMGT unique numbering for C-domain [47] (by comparison to the V-domain IMGT unique numbering [48]) are underlined. Alias variant names found in the literature are written in blue in column 4 'Amino Acid Changes with the Eu Positions'. The background color indicates a reduction (pink color) or an enhancement (green color) of the involved effector 'Property and Function'. For other 'Property and Function', background colors refer to structure (yellow), half-life (pale blue color) or protein A (pale orange).

IMGT nomenclature, Eu positions and IMGT motif of engineered Fc variants involved in:
The property and function type is indicated by a number from 1 to 19 with references to the separate tables per type of the manuscript.
1. antibody-dependent cellular cytotoxicity (ADCC) reductio (Table 5) 2. antibody-dependent cellular cytotoxicity (ADCC) enhancement (Table 6).
3. antibody-dependent cellular cytotoxicity (ADCC) and antibody-dependent cellular phagocytosis (ADCP) enhancement (Table 7).
4. complement-dependent cytotoxicity (CDC) enhancement (Table 8)
5. complement-dependent cytotoxicity (CDC) reduction (Table 9).
6. antibody-dependent cellular cytotoxicity (ADCC) and complement-dependent cytotoxicity (CDC) reduction (Table 10).
7. B cell inhibition by coengagement of antigen and FcγR on same cell (Table 11).
8. knock out CH2 84.4 glycosylation (Table 12)
9. half-life increase or decrease (Table 13)
10. abrogation of binding to Protein A (Table 14)
11. formation of additional bridge stabilizing CH2 in the absence of N84.4 (Eu 297) glycosylation (Table 15).
12. prevention of IgG4 half-IG exchange (Table 16).
13. hexamerisation (Table 17).
14. knobs-into-holes and enhancement of heteropairing H-H of bispecific anti (Table 18). 15. suppression of inter H-L and/or inter H-H disulfide bridges (Table 19).
16. site-specific drug attachment (Table 20).
17. enhancement of hetero pairing (Table 21). 18. control of half-IG exchange of bispecific IgG4.(Table 22).
19. reducing acid-induced aggregation (Table 23).

Amino acids: Amino acids are shown in the one-letter abbreviation [49]. Underlined amino acids in the 'Motif' column correspond to additional positions in the IMGT unique numbering for C-domain [47,50–52]

Species:
Homsap: Homo sapiens

Canlupfam: Canis lupus familiaris
Musmus: Mus musculus

IMGT engineered Fc variant name

G1v1: immunoglobulin gamma 1 heavy chain constant region engineered variant 1
Homsap
G2v1: immunoglobulin gamma 2 heavy chain constant region engineered variant 1
G3v1: : immunoglobulin gamma 3 heavy chain constant region engineered variant 1
G4v1: : immunoglobulin gamma 4 heavy chain constant region engineered variant 1
KCv: immunoglobulin kappa light chain constant region engineered variant
LCv: immunoglobulin lambda light chain constant region engineered variant

References

1. Edelman, G.M.; Cunningham, B.A.; Gall, W.E.; Gottlieb, P.D.; Rutishauser, U.; Waxdal, M.J. The covalent structure of an entire gammaG immunoglobulin molecule. *Proc. Natl. Acad. Sci. USA* **1969**, *63*, 78–85. https://doi.org/10.1073/pnas.63.1.78.
2. Kabat, E.A.; Wu, T.T.; Reid-Miller, M.; Perry, H.M.; Gottesman, K.S. *Sequences of Proteins of Immunological Interest*, 5th ed.; NIH publication n° 91-3242; US Department of Health and Human Services: Washington, DC, USA, 1991; pp. 662, 680, 689.
3. Chappel, M.S.; Isenman, D.E.; Everett, M.; Xu, Y.Y.; Dorrington, K.J.; Klein, M.H. Identification of the Fc gamma receptor class I binding site in human IgG through the use of recombinant IgG1/IgG2 hybrid and point-mutated antibodies. *Proc. Natl. Acad. Sci. USA* **1991**, *88*, 9036–9040. https://doi.org/10.1073/pnas.88.20.9036.
4. Idusogie, E.E.; Presta, L.G.; Gazzano-Santoro, H.; Totpal, K.; Wong, P.Y.; Ultsch, M.; Meng, Y.G.; Mulkerrin, M.G. Mapping of the C1q binding site on rituxan, a chimeric antibody with a human IgG1 Fc. *J. Immunol.* **2000**, *164*, 4178–4184. https://doi.org/10.4049/jimmunol.164.8.4178.
5. Idusogie, E.E.; Wong, P.Y.; Presta, L.G.; Gazzano-Santoro, H.; Totpal, K.; Ultsch, M.; Mulkerrin, M.G. Engineered antibodies with increased activity to recruit complement. *J. Immunol.* **2001**, *166*, 2571–2575. https://doi.org/10.4049/jimmunol.166.4.2571.
6. Shields, R.L.; Namenuk, A.K.; Hong, K.; Meng, Y.G.; Rae, J.; Briggs, J.; Xie, D.; Lai, J.; Stadlen, A.; Li, B.; et al. High resolution mapping of the binding site on human IgG1 for Fc gamma RI, Fc gamma RII, Fc gamma RIII, and FcRn and design of IgG1 variants with improved binding to the Fc gamma R. *J. Biol. Chem.* **2001**, *276*, 6591–6604. https://doi.org/10.1074/jbc.M009483200.
7. Lazar, G.A.; Dang, W.; Karki, S.; Vafa, O.; Peng, J.S.; Hyun, L.; Chan, C.; Chung, H.S.; Eivazi, A.; Yoder, S.C.; et al. Engineered antibody Fc variants with enhanced effector function. *Proc. Natl. Acad. Sci. USA* **2006**, *103*, 4005–4010. https://doi.org/10.1073/pnas.0508123103.
8. Oganesyan, V.; Damschroder, M.M.; Leach, W.; Wu, H.; Dall'Acqua, W.F. Structural characterization of a mutated, ADCC-enhanced human Fc fragment. *Mol. Immunol*. **2008**, *45*, 1872–1882. https://doi.org/10.1016/j.molimm.2007.10.042.
9. Stavenhagen, J.B.; Gorlatov, S.; Tuaillon, N.; Rankin, C.T.; Li, H.; Burke, S.; Huang, L.; Vijh, S.; Johnson, S.; Bonvini, E.; et al. Fc optimization of therapeutic antibodies enhances their ability to kill tumor cells in vitro and controls tumor expansion in vivo via low-affinity activating Fcgamma receptors. *Cancer Res.* **2007**, *67*, 8882–8890. https://doi.org/10.1158/0008-5472.CAN-07-0696.
10. Mimoto, F.; Igawa, T.; Kuramochi, T.; Katada, H.; Kadono, S.; Kamikawa, T.; Shida-Kawazoe, M.; Hattori, K. Novel asymmetrically engineered antibody Fc variant with superior FcγR binding affinity and specificity compared with afucosylated Fc variant. *mAbs* **2013**, *5*, 229–236. https://doi.org/10.4161/mabs.23452.
11. Ahmed, A.A.; Keremane, S.R.; Vielmetter, J.; Bjorkman, P.J. Structural characterization of GASDALIE Fc bound to the activating Fc receptor FcγRIIIa. *J. Struct. Biol*. **2016**, *1*, 78–89. https://doi.org/10.1016/j.jsb.2016.02.001.

12. Richards, J.O.; Karki, S.; Lazar, G.A.; Chen, H.; Dang, W.; Desjarlais, J.R. Optimization of antibody binding to FcgammaRIIa enhances macrophage phagocytosis of tumor cells. *Mol. Cancer Ther*. **2008**, *7*, 2517–2527. https://doi.org/10.1158/1535-7163.MCT-08-0201.
13. Xu, D.; Alegre, M.L.; Varga, S.S.; Rothermel, A.L.; Collins, A.M.; Pulito, V.L.; Hanna, L.S.; Dolan, K.P.; Parren, P.W.; Bluestone, J.A.; et al. In vitro characterization of five humanized OKT3 effector function variant antibodies. *Cell Immunol.* **2000**, *200*, 16–26. https://doi.org/10.1006/cimm.2000.1617.
14. Hezareh, M.; Hessell, A.J.; Jensen, R.C.; van de Winkel, J.G.; Parren, P.W. Effector function activities of a panel of mutants of a broadly neutralizing antibody against human immunodeficiency virus type 1. *J. Virol*. **2001**, *75*, 12161–12168. https://doi.org/10.1128/JVI.75.24.12161-12168.2001.
15. Schlothauer, T.; Herter, S.; Koller, C.F.; Grau-Richards, S.; Steinhart, V.; Spick, C.; Kubbies, M.; Klein, C.; Umaña, P.; Mössner, E. Novel human IgG1 and IgG4 Fc-engineered antibodies with completely abolished immune effector functions. *Protein Eng. Des. Sel.* 2016, *29*, 457–466. https://doi.org/10.1093/protein/gzw040.
16. Moore, G.L.; Chen, H.; Karki, S.; Lazar, G.A. Engineered Fc variant antibodies with enhanced ability to recruit complement and mediate effector functions. *mAbs* **2010**, *2*, 181–189. https://doi.org/10.4161/mabs.2.2.11158.
17. Diebolder, C.A.; Beurskens, F.J.; de Jong, R.N.; Koning, R.I.; Strumane, K.; Lindorfer, M.A.; Voorhorst, M.; Ugurlar, D.; Rosati, S.; Heck, A.J.; et al. Complement is activated by IgG hexamers assembled at the cell surface. *Science* **2014**, *343*,1260–1263. https://doi.org/10.1126/science.1248943.
18. Robbie, G.J.; Criste, R.; Dall'acqua, W.F.; Jensen, K.; Patel, N.K.; Losonsky, G.A.; Griffin, M.P. A novel investigational Fc-modified humanized monoclonal antibody, motavizumab-YTE, has an extended half-life in healthy adults. *Antimicrob. Agents Chemother*. **2013**, *57*, 6147–6153. https://doi.org/10.1128/AAC.01285-13.
19. Dall'Acqua, W.F.; Woods, R.M.; Ward, E.S.; Palaszynski, S.R.; Patel, N.K.; Brewah, Y.A.; Wu, H.; Kiener, P.A.; Langermann, S. Increasing the affinity of a human IgG1 for the neonatal Fc receptor: Biological consequences. *J. Immunol.* **2002**, *169*, 5171–5180. https://doi.org/10.4049/jimmunol.169.9.5171.
20. Alegre, M.L.; Collins, A.M.; Pulito, V.L.; Brosius, R.A.; Olson, W.C.; Zivin, R.A.; Knowles, R.; Thistlethwaite, J.R.; Jolliffe, L.K.; Bluestone, J.A. Effect of a single amino acid mutation on the activating and immunosuppressive properties of a "humanized" OKT3 monoclonal antibody. *J. Immunol*. **1992**, 148, 3461–3468.
21. Zalevsky, J.; Chamberlain, A.K.; Horton, H.M.; Karki, S.; Leung, I.W.; Sproule, T.J.; Lazar, G.A.; Roopenian, D.C.; Desjarlais, J.R. Enhanced antibody half-life improves in vivo activity. *Nat. Biotechnol*. **2010**, *28*, 157–159. https://doi.org/10.1038/nbt.1601
22. Chu, S.Y.; Vostiar, I.; Karki, S.; Moore, G.L.; Lazar, G.A.; Pong, E.; Joyce, P.F.; Szymkowski, D.E.; Desjarlais, J.R. Inhibition of B cell receptor-mediated activation of primary human B cells by coengagement of CD19 and FcgammaRIIb with Fc-engineered antibodies. *Mol. Immunol.* **2008**, *45*, 3926–3933. https://doi.org/10.1016/j.molimm.2008.06.027.
23. Szili, D.; Cserhalmi, M.; Bankó, Z.; Nagy, G.; Szymkowski, D.E.; Sármay, G. Suppression of innate and adaptive B cell activation pathways by antibody coengagement of FcγRIIb and CD19. *mAbs* **2014**, *6*, 991–999. https://doi.org/10.4161/mabs.28841.
24. Ridgway, J.B.; Presta, L.G.; Carter, P. 'Knobs-into-holes' engineering of antibody CH3 domains for heavy chain heterodimerization. *Protein Eng*. **1996**, *9*, 617–621. https://doi.org/10.1093/protein/9.7.617.
25. Leabman, M.K.; Meng, Y.G.; Kelley, R.F.; DeForge, L.E.; Cowan, K.J.; Iyer, S. Effects of altered FcγR binding on antibody pharmacokinetics in cynomolgus monkeys. *mAbs* 2013, *5*, 896–903. https://doi.org/10.4161/mabs.26436.
26. Smith, P.; DiLillo, D.J.; Bournazos, S.; Li, F.; Ravetch, J.V. Mouse model recapitulating human Fcγ receptor structural and functional diversity. *Proc. Natl. Acad. Sci. USA* **2012**, *109*, 6181–6186. https://doi.org/10.1073/pnas.1203954109.
27. Shang, L.; Daubeuf, B.; Triantafilou, M.; Olden, R.; Dépis, F.; Raby, AC.; Herren, S.; Dos Santos, A.; Malinge, P.; Dunn-Siegrist, I.; et al. Selective antibody intervention of Toll-like receptor 4 activation through Fc γ receptor tethering. *J. Biol. Chem*. **2014**, *289*, 15309–15318. https://doi.org/10.1074/jbc.M113.537936.
28. Vafa, O.; Gilliland, G.L.; Brezski, R.J.; Strake, B.; Wilkinson, T.; Lacy, E.R.; Scallon, B.; Teplyakov, A.; Malia, T.J.; Strohl, W.R.; An engineered Fc variant of an IgG eliminates all immune effector functions via structural perturbations. *Methods* **2014**, *65*, 114–126. https://doi.org/10.1016/j.ymeth.2013.06.035.
29. Brinkhaus, M.; Douwes, R.G.J.; Bentlage, A.E.H.; Temming, A.R.; de Taeye, S.W.; Tammes Buirs, M.; Gerritsen, J.; Mok, J.Y.; Brasser, G.; Ligthart, P.C.; et al**.** Glycine 236 in the lower hinge region of human IgG1 differentiates FcγR from Complement effector function. *J. Immunol*. **2020**, *205*, 3456–3467. https://doi.org/10.4049/jimmunol.2000961.
30. Wilkinson, I.; Anderson, S.; Fry, J.; Julien, L.A.; Neville, D.; Qureshi, O.; Watts, G.; Hale, G. Fc-engineered antibodies with immune effector functions completely abolished. *PLoS ONE* 2021, *16*, e0260954. https://doi.org/10.1371/journal.pone.0260954.
31. Borrok, M.J.; Mody, N.; Lu, X.; Kuhn, M.L.; Wu, H.; Dall'Acqua, W.F.; Tsui, P. An "Fc-Silenced" IgG1 format with extended half-life designed for improved stability. *J. Pharm. Sci*. **2017**, 106, 1008–1017. https://doi.org/10.1016/j.xphs.2016.12.023.

32. Lefranc, M.-P. WHO-IUIS Nomenclature Subcommittee for Immunoglobulins and T cell receptors report. *Immunogenetics* **2007**, *59*, 899–902.
33. Natsume, A.; In, M.; Takamura, H.; Nakagawa, T.; Shiz, Y.; Kitajima, K.; Wakitani, M.; Ohta, S.; Satoh, M.; Shitara, K.; et al. Engineered antibodies of IgG1/IgG3 mixed isotype with enhanced cytotoxic activities. *Cancer Res.* **2008**, *68*, 3863–3872. https://doi.org/10.1158/0008-5472.CAN-07-6297.
34. An, Z.; Forrest, G.; Moore, R.; Cukan, M.; Haytko, P.; Huang, L.; Vitelli, S.; Zhao, J.Z.; Lu, P.; Hua, J.; et al. IgG2m4, an engineered antibody isotype with reduced Fc function. *mAbs* **2009**, *1*, 572–579. https://doi.org/10.4161/mabs.1.6.10185.
35. Hinton, P.R.; Johlfs, M.G.; Xiong, J.M.; Hanestad, K.; Ong, K.C.; Bullock, C.; Keller, S.; Tang, M.T.; Tso, J.Y.; Vásquez, M.; et al. Engineered human IgG antibodies with longer serum half-lives in primates. *J. Biol. Chem*. **2004**, *279*, 6213–6216. https://doi.org/10.1074/jbc.C300470200.
36. Saito, S.; Namisaki, H.; Hiraishi, K.; Takahashi, N.; Iida, S. Engineering a human IgG2 antibody stable at low pH. *Protein Sci*. **2020**, *29*, 1186–1195. https://doi.org/10.1002/pro.3852.
37. Oganesyan, V.; Damschroder, M.M.; Cook, K.E.; Li, Q.; Gao, C.; Wu, H.; Dall'Acqua, W.F. Structural insights into neonatal Fc receptor-based recycling mechanisms. *J. Biol. Chem.* **2014**, *289*, 7812–7824. https://doi.org/10.1074/jbc.M113.537563.
38. Rother, R.P.; Rollins, S.A.; Mojcik, C.F.; Brodsky, R.A.; Bell, L. Discovery and development of the complement inhibitor eculizumab for the treatment of paroxysmal nocturnal hemoglobinuria. *Nat. Biotechnol*. **2007**, *25*, 1256–1264. https://doi.org/10.1038/nbt1344.
39. Stapleton, N.M.; Andersen, J.T.; Stemerding, A.M.; Bjarnarson, S.P.; Verheul, R.C.; Gerritsen, J.; Zhao, Y.; Kleijer, M.; Sandlie, I.; de Haas, M.; et al. Competition for FcRn-mediated transport gives rise to short half-life of human IgG3 and offers therapeutic potential. *Nat. Commun.* **2011**, 2, 599. https://doi.org/10.1038/ncomms1608.
40. Tao, M.H.; Smith, R.I.; Morrison, S.L. Structural features of human immunoglobulin G that determine isotype-specific differences in complement activation. *J. Exp. Med*. **1993**, *178*, 661–667. https://doi.org/10.1084/jem.178.2.661.
41. Labrijn, A.F.; Buijsse, A.O.; van den Bremer, E.T.; Verwilligen, A.Y.; Bleeker, W.K.; Thorpe, S.J.; Killestein, J.; Polman, C.H.; Aalberse, R.C.; Schuurman, J.; et al. Therapeutic IgG4 antibodies engage in Fab-arm exchange with endogenous human IgG4 in vivo. *Nat. Biotechnol*. **2009**, *27*, 767–771. https://doi.org/10.1038/nbt.1553.
42. Labrijn, A.F.; Rispens, T.; Meesters, J.; Rose, R.J.; den Bleker, T.H.; Loverix, S.; van den Bremer, E.T.; Neijssen, J.; Vink, T.; Lasters, I.; et al. Species-specific determinants in the IgG CH3 domain enable Fab-arm exchange by affecting the noncovalent CH3-CH3 interaction strength. *J. Immunol*. **2011**, *187*, 3238–3246. https://doi.org/10.4049/jimmunol.1003336.
43. Lefranc, M.-P.; Lefranc, G. *The Immunoglobulin FactsBook*; Academic Press: London, UK, 2001; pp. 1–457.
44. Zhang, J.; Huang, Y.; Xi, G.; Zhang, F. HX008: A humanized PD-1 blocking antibody with potent antitumor activity and superior pharmacologic properties. *mAbs* **2020**, *12*, 1724751. https://doi.org/10.1080/19420862.2020.1724751.
45. Duncan, A.R.; Woof, J.M.; Partridge, L.J.; Burton, D.R.; Winter, G. Localization of the binding site for the human high-affinity Fc receptor on IgG. *Nature* **1988**, *332*, 563–564. https://doi.org/10.1038/332563a0.
46. Duncan, A.R.; Winter, G. The binding site for C1q on IgG. *Nature* **1988**, *332*, 738–740. https://doi.org/10.1038/332738a0.
47. Lefranc, M.-P.; Pommié, C.; Kaas, Q.; Duprat, E.; Bosc, N.; Guiraudou, D.; Jean, C.; Ruiz, M.; Da Piedade, I.; Rouard, M.; et al. IMGT unique numbering for immunoglobulin and T cell receptor constant domains and Ig superfamily C-like domains. *Dev. Comp. Immunol.* **2005**, *29*, 185–203.
48. Lefranc, M.-P.; Pommié, C.; Ruiz, M.; Giudicelli, V.; Foulquier, E.; Truong, L.; Thouvenin-Contet, V.; Lefranc, G. IMGT unique numbering for immunoglobulin and T cell receptor variable domains and Ig superfamily V-like domains. *Dev. Comp. Immunol.* **2003**, *27*, 55–77.
49. Pommié, C.; Levadoux, S.; Sabatier, R.; Lefranc, G.; Lefranc, M.-P. IMGT standardized criteria for statistical analysis of immunoglobulin V-REGION amino acid properties. *J. Mol. Recognit.* **2004**, *17*, 17–32.
50. Lefranc, M.-P. IMGT unique numbering for the Variable (V), Constant (C), and Groove (G) domains of IG, TR, MH, IgSF, and MhSF. *Cold Spring Harb. Protoc.* **2011**, *6*, 633–642. https://doi.org/10.1101/pdb.ip85.
51. Lefranc, M.-P. IMGT unique numbering. In *Encyclopedia of Systems Biology*; Dubitzky, W., Wolkenhauer, O., Cho, K.-H., Yokota, H., Eds.; Springer: New York, NY, USA, 2013; pp. 952–959. https://doi.org/10.1007/978-1-4419-9863-7_127.
52. Lefranc, M-P. Immunoinformatics of the V, C, and G domains: IMGT® definitive system for IG, TR and IgSF, MH, and MhSF. In *Immunoinformatics: From Biology to Informatics*, 2nd ed.; De, R.K., Tomar, N., Eds.; Methods in Molecular Biology' Humana Press: New Yor, NY, USA, 2014; Volume 1184, pp. 59–107. https://doi.org/10.1007/978-1-4939-1115-8_4.
